# HSPA8 Activates Wnt/β‐Catenin Signaling to Facilitate BRAF V600E Colorectal Cancer Progression by CMA‐Mediated CAV1 Degradation

**DOI:** 10.1002/advs.202306535

**Published:** 2023-11-16

**Authors:** Bowen Li, Hui Ming, Siyuan Qin, Li Zhou, Zhao Huang, Ping Jin, Liyuan Peng, Maochao Luo, Tingting Zhang, Kui Wang, Rui Liu, Yih‐Cherng Liou, Edouard C. Nice, Jingwen Jiang, Canhua Huang

**Affiliations:** ^1^ State Key Laboratory of Biotherapy and Cancer Center West China Hospital and West China School of Basic Medical Sciences and Forensic Medicine Sichuan University and Collaborative Innovation Center for Biotherapy Chengdu 610041 P. R. China; ^2^ State Key Laboratory of Oral Diseases National Clinical Research Center for Oral Diseases Chinese Academy of Medical Sciences Research Unit of Oral Carcinogenesis and Management West China Hospital of Stomatology Sichuan University Chengdu Sichuan 610041 P. R. China; ^3^ Department of Biological Sciences Faculty of Science National University of Singapore Singapore 117543 Singapore; ^4^ Graduate School for Integrative Sciences and Engineering National University of Singapore Singapore 117573 Singapore; ^5^ Department of Biochemistry and Molecular Biology Monash University Clayton VIC 3800 Australia; ^6^ West China School of Public Health and West China Fourth Hospital Sichuan University Chengdu 610041 P. R. China

**Keywords:** BRAF V600E, drug resistance, epithelial–mesenchymal transition, heat shock 70 kDa protein 8, Wnt/β‐catenin

## Abstract

BRAF V600E attracts wide attention in the treatment of colorectal cancer (CRC) as stratifying and predicting a refractory classification of CRC. Recent evidence indicates that Wnt/β‐catenin signaling is broadly activated and participates in the refractoriness of BRAF V600E CRC, but the underlying molecular mechanism needs to be elucidated. Here, heat shock 70 kDa protein 8 (HSPA8), an essential regulator in chaperone‐mediated autophagy (CMA), is identified as a potential therapeutic target for advanced BRAF V600E CRC. These results show that HSPA8 is transcriptionally upregulated in BRAF V600E CRC, which promotes CMA‐dependent degradation of caveolin‐1 (CAV1) to release β‐catenin into the nucleus and thus activates the Wnt/β‐catenin pathway, contributing to metastasis and progression of BRAF V600E CRC. Of note, HSPA8 directly interacts with the KIFSN motif on CAV1, the interaction can be enhanced by p38 MAPK‐mediated CAV1 S168 phosphorylation. Furthermore, pharmacological targeting HSPA8 by VER155008 exhibits synergistic effects with BRAF inhibitors on CRC mouse models. In summary, these findings discover the important role of the HSPA8/CAV1/β‐catenin axis in the development of refractory BRAF V600E CRC and highlight HSPA8 as a predictive biomarker and therapeutic target in clinical practice.

## Graphical abstract

Upregulated heat shock 70 kDa protein 8 (HSPA8) predicts poor prognosis of BRAF V600E colorectal cancer. HSPA8 interacts with Caveolin‐1 at the KIFSN motif, leading to Caveolin‐1 degradation and the subsequent β‐catenin nuclear translocation. The Wnt/β‐catenin pathway is then activated to promote metastasis. Notably, HSPA8 inhibitor VER155008 sensitizes BRAF V600E colorectal cancer to targeted drugs, which offers potential therapeutic strategies.

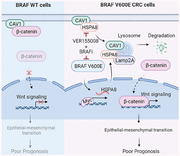

## 1 Introduction

Colorectal cancer (CRC) is the second most deadly cancer worldwide (over 0.93 million in 2020), according to statistics from the World Health Organization.^[^ ^1^ ^]^ Despite favorable responses to surgery and chemotherapy in early‐stage CRC, patients with advanced CRC lack effective clinical interventions, exhibiting a 5 years survival rate below 14%.^[^ ^2^ ^]^ BRAF V600E mutation (which occurs in ≈12% of metastatic CRC patients) is considered an essential prognostic marker of metastatic CRC, which results in failure of standard chemotherapy and only ≈11 months median overall survival.^[^ ^3^ ^]^ Although BRAF V600E‐specific inhibitors (e.g., Dabrafenib, Encorafenib, and Agerafenib) show up to 80% response rates in melanoma, the response rate is only 5% in CRC, and the underlying molecular mechanism is still unclear.^[^ ^4^ ^]^ The low response rate of BRAF V600E patients to targeted drugs may be due to the intrinsic bypass activation of downstream MAPK cascades, including the EGFR‐CRAF‐MEK pathway.^[^ ^5^ ^]^ Besides, extrinsic activation of other oncogenic pathways (e.g., the PI3K/Akt pathway) may also lead to unresponsiveness.^[^ ^6^ ^]^ The combinational use of BRAF and MEK inhibitors;^[^ ^7^ ^]^ BRAF, EGFR, and MEK inhibitors;^[^ ^8^ ^]^ BRAF and PI3K inhibitors;^[^ ^9^ ^]^ and BRAF, PI3K, and EGFR inhibitors^[^ ^9^ ^]^ have achieved 12%, 21%, 23%, and 32% response rates, respectively. These encouraging developments motivate the exploration of additional molecular mechanisms responsible for the poor prognosis of BRAF V600E CRC patients. Recent studies indicate that aberrant activation of Wnt/β‐catenin signaling, one of the essential features in the development and occurrence of CRC,^[^ ^10^ ^]^ is closely related to the refractoriness of BRAF V600E CRC.^[^ ^11^ ^]^ However, the underlying molecular basis linking Wnt/β‐catenin signaling and BRAF V600E‐mediated chemoresistance warrant further investigation.

Heat shock 70 kDa protein 8 (HSPA8 or HSC70), the main housekeeping protein of the heat shock protein 70 family, is considered an essential chaperone molecule in assisting protein folding surveillance and chaperone‐mediated autophagy (CMA), which facilitates the degradation of selected proteins.^[^ ^12^ ^]^ Owing to its biological function, HSPA8 has been recognized as a therapeutic target for autoimmune diseases.^[^ ^13^ ^]^ Recent studies have shown that protein degradation, mainly through the ubiquitin–proteasome system and autophagy‐lysosome system, plays an important role in maintaining cellular homeostasis, and its dysregulation is closely related to tumorigenesis and tumor progression.^[^ ^14^ ^]^ As a pivotal regulator of CMA, HSPA8 is upregulated in various cancer types and considered a predictive biomarker.^[^ ^15^ ^]^ In addition, it has been revealed that HSPA8 is closely correlated with therapeutic response in several cancer types treated with various drug regimens, suggesting HSPA8 may have potential clinical utility as a promising drug target.^[^ ^16^ ^]^

In this study, we find the upregulation of HSPA8 as a potential cause of poor prognosis in BRAF V600E CRC patients, which activates the Wnt/β‐catenin signaling pathway through a caveolin‐1 (CAV1)‐dependent mechanism. Recent studies indicate that CAV1 can prevent the translocation of β‐catenin into the nucleus by trapping β‐catenin in the cytoplasm.^[^ ^17^ ^]^ HSPA8 interacts with CAV1 through the KIFSN motif and promotes CMA‐dependent degradation of CAV1, thus releasing β‐catenin into the nucleus and activating the Wnt pathway. In addition, VER155008, a small molecule inhibitor of HSPA8, sensitizes BRAF V600E CRC cells to BRAF inhibitors. Our results report the underlying mechanisms of HSPA8‐mediated drug resistance to BRAF inhibitors in CRC and highlight HSPA8 as a potential biomarker and therapeutic target for BRAF V600E CRC patients.

## 2 Results

### 2.1 BRAF V600E Mutation Induces HSPA8 Expression in Human CRC

**Figure 1:**
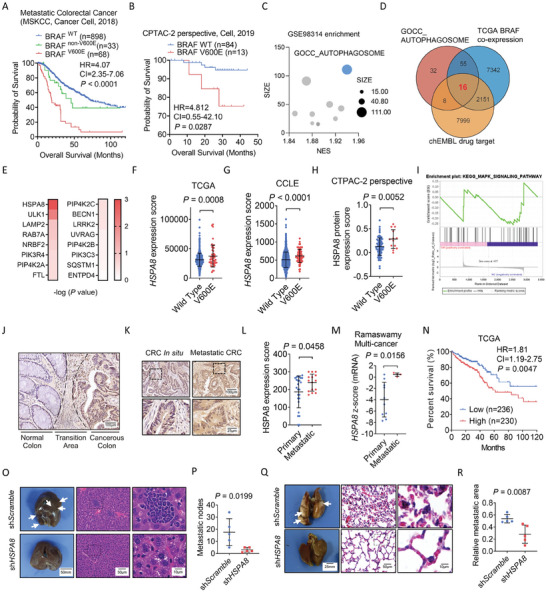
Elevated HSPA8 expression in human colorectal cancer with BRAF V600E mutation. A) Overall survival of CRC patients with no mutation, V600E mutation, or other mutations in BRAF according to a metastatic colorectal cancer dataset (MSKCC, Cancer cell, 2018). B) Overall survival of CRC patients with no mutation or V600E mutation in BRAF according to the CTPAC‐2 perspective dataset. C) Gene set enrichment analysis (GSEA) of the GEO dataset GSE98314 between groups with and without the treatment of BRAF inhibitor using the GOCC enrichment. The vertical axis represents the gene set size, and the horizontal represents normalized enrichment score (NES) (The top ten enriched pathways are represented in a scatter plot). D) Venn group designations showing that 16 genes were enriched in the GSE98314 GOCC_AUTOPHAGOSOME gene set, TCGA BRAF co‐expression genes, and chEMBL drug target gene set. E) Evaluation of the candidate genes involved in BRAF V600E targeted drug treatment based on dataset GSE98314. F–H) HSPA8 mRNA levels in CRC patients with or without BRAF V600E mutation according to TCGA, CCLE, or CTPAC‐2 perspective dataset (Student's *t* test). I) Gene set enrichment analysis (GSEA) of the KEGG MAPK signaling pathway was performed in the RKO and RKO shHSPA8 groups. J) Representative images of HSPA8 immunohistochemical staining in the normal colon (adjacent tissue), transition area, and cancerous colon. Scale bar: 100 µm. K) Representative images of HSPA8 immunohistochemical staining in primary (*n* = 25) or metastatic sites (*n* = 15) of CRC. Scale bar: 100 µm (above), 25 µm (below). L) Statistical quantification of HSPA8 immunohistochemical staining in primary or metastatic sites of CRC (*P* = 0.0458, Student's *t* test). M) HSPA8 mRNA levels in primary or metastatic sites of CRC patients according to the Oncomine dataset Ramaswamy Multi‐cancer (*P* = 0.0156, Student's *t* test). N) Overall survival of CRC patients according to the HSPA8 mRNA levels in TCGA dataset (*P* = 0.0047, log‐rank [Mantel‐Cox] test). O) Representative images of liver metastatic nodules and H&E staining of RKO (BRAF V600E)‐derived orthotopic colorectal cancer model. Scale bar: 50 mm (left), 50 µm (medium), 10 µm (right). P) Statistical quantification of liver metastatic nodule number. Q) Representative images of lung metastatic area and H&E staining of mouse tissues. Scale bar: 25 mm (left), 50 µm (medium), 10 µm (right). R) Statistical quantification of lung metastatic area of the tail vein injection mice model.

Recent studies indicate that the BRAF V600E mutation is an essential prognostic and predictive biomarker for metastatic CRC patients, with which patients may have a worse prognosis even after BRAF‐targeted therapy (**Figure** **1A,B**).^[^ ^10^, ^18^ ^]^ Recent studies indicate that several pro‐survival pathways (e.g., PI3K pathway, autophagy, and metabolism related pathways) may participate in the low response to BRAF‐targeted therapeutics.^[^ ^19^ ^]^ To investigate the underlying molecular mechanisms, we analyzed a dataset (GSE98314) containing discrepant gene expression in tumor cells with or without the treatment of BRAF V600E inhibitor Dabrafenib through gene set enrichment analysis (GSEA), which suggested that the autophagosome gene set (111 genes) might play a pivotal role in response to the BRAF‐targeted therapy (Figure 1C). Subsequently, the co‐expressed genes with BRAF were examined in the TCGA database, and 71 overlapped genes were identified. To screen druggable targets for clinical treatment, the 71 candidates were overlapped with the ChEMBL database (Figure 1D). The expression levels of the selected 16 candidate genes are listed, among which HSPA8 was the most significant gene (Figure 1E). Based on clinical data from the public database, HSPA8 was upregulated in patients harboring BRAF missense mutation (Figure S1A,B, Supporting Information), especially in patients with BRAF V600E mutation (Figure 1F–H). Consistently, GSEA enrichment indicated that the BRAF downstream signaling MAPK pathway was significantly downregulated in the HSPA8 depletion group (Figure 1I). To further explore the BRAF V600E‐mediated upregulation of HSPA8, we exogenously expressed WT BRAF and BRAF V600E mutant protein and examined the expression levels of HSPA8 and BRAF downstream transcription factors, including c‐Myc, c‐FOS, and c‐JUN, that may regulate the expression of HSPA8 (Figure S1C–H, Supporting Information).^[^ ^20^ ^]^ Notably, the expression levels of these transcription factors were not obviously changed, while BRAF V600E increased the binding ratio of c‐Myc and the promotor of HSPA8 according to the ChIP‐qPCR assay (Figure S1I, Supporting Information). Besides, the HSPA8 levels were upregulated when overexpression c‐Myc (Figure S1J,K, Supporting Information), and the expression of HSPA8 and c‐Myc was positively correlated according to the databases (Figure S1L,M, Supporting Information), indicating that c‐Myc might be responsible for the upregulated HSPA8 expression in BRAF V600E cells. To further evaluate the role of HSPA8 in tumorigenesis and tumor development in CRC, we then evaluated the protein level of HSPA8 in 25 CRC tissues compared to adjacent normal tissues. Immunohistochemical analysis showed an increased expression of HSPA8 in CRC tissues (Figure S2A,B, Supporting Information). Consistently, increased HSPA8 mRNA expression in CRC patients was also observed by the analysis of the TCGA and GSE20916 datasets (Figure S2C,D, Supporting Information). In addition, the western blot indicated a significant increase of HSPA8 in CRC tissues (Figure S2E,F, Supporting Information). Metastasis of late‐stage CRC is considered one of the leading causes of the poor prognosis of patients harboring the BRAF V600E mutation.^[^ ^21^ ^]^ Indeed, HSPA8 expression was significantly increased in metastatic tissues, which was highly related to the poor prognosis of CRC patients (Figure 1J–N). Consistently, a positive correlation was observed between the HSPA8 protein level and migratory ability of several CRC cell lines, including HCT116, HT29, SW480, SW48, SW620, RKO, and LOVO (Figure S2G–K, Supporting Information), among which the genotype of RKO and HT29 is BRAF V600E. RKO expressed higher HSPA8 level, while HT29 showed relatively lower HSPA8 level. Hence, we decided to perform HSPA8 overexpression in HT29 cell line and KD experiments in RKO cell line. Moreover, HSPA8 depletion attenuated CRC metastasis in both orthotropic and tail vein injection models (Figure 1O–R and Figure S2L,M, Supporting Information). Taken together, our data suggests that the BRAF V600E mutation could upregulate HSPA8 and HSPA8 predicts poor prognosis of CRC patients.

To verify the role of HSPA8 in the response of CRC cells to BRAF V600E inhibitor, we assessed the IC_50_ of BRAF V600E inhibitor in different CRC cell lines. Notably, the IC_50_ of BRAF inhibitors showed a positive correlation with HSPA8 expression in BRAF V600E cell lines but not in WT cell lines (Figure S3A,B, Supporting Information). In addition, HSPA8 was upregulated in HT29 and RKO cell lines (both with BRAF V600E mutation) after Dabrafenib treatment (Figure S3C, Supporting Information). The different response rates to BRAF V600E‐targeted drugs between BRAF V600E‐mutated melanoma and CRC, and the differential expression pattern of HSPA8 and MYC were consistent with our findings (Figure S3D–F, Supporting Information). Knockdown of HSPA8 sensitized RKO cells to Dabrafenib treatment (Figure S3G, Supporting Information), indicating the essential role of HSPA8 in the BRAF inhibitor response, and targeting HSPA8 may facilitate the efficacy of BRAF V600E inhibitor.

### 2.2 HSPA8 Promotes Epithelial–Mesenchymal Transition in BRAF V600E Colorectal Cancer Cells

**Figure 2:**
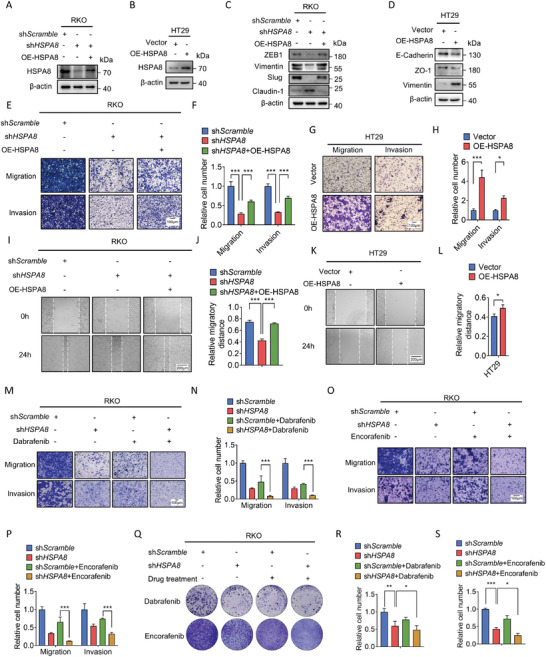
HSPA8 promotes epithelial‐mesenchymal transition in BRAF V600E CRC cells. A) Immunoblotting analysis of RKO cells stably expressing shScramble, shHSPA8, or shHSPA8+OE‐HSPA8. B) Immunoblotting analysis of HT29 cells stably expressing vector or HSPA8. C,D) Immunoblotting analysis of the effects of HSPA8 on the expression of EMT marker proteins in RKO and HT29 cells. E–H) Transwell assay showing the migration and invasion ability of RKO cells transfected with shScramble, shHSPA8, or shHSPA8+OE‐HSPA8 and HT29 cells stably expressing vector or HSPA8. Scale bar: 100 µm. I–L) Wound healing assay showing the migration of RKO cells transfected with shScramble, shHSPA8, or shHSPA8+OE‐HSPA8 and HT29 cells stably expressing vector or HSPA8 after 24 h. Scale bar: 200 µm. M–P) Transwell assays showing the effects of HSPA8 and Dabrafenib/Encorafenib on cell migration and invasion. Scale bar: 100 µm. Q–S) Representative images of the colony formation of the indicated cells and quantification of clone numbers. ***P < 0.001, **P < 0.01, *P < 0.05, and data are the mean ± SEM from at least three independent experiments.

To investigate the function of HSPA8 in BRAF V600E CRC, endogenous HSPA8 was stably knocked down by short hairpin RNA (shRNA) in RKO (BRAF V600E cell line) and SW480 (BRAF wild‐type cell line) cells (**Figure** **2**A and Figure S4A, Supporting Information). Exogenous HSPA8 was overexpressed in HT29 (BRAF V600E cell line) (Figure 2B). The results showed that HSPA8 knockdown suppressed the expression of mesenchymal markers (ZEB1, Vimentin, and Slug), upregulated the epithelial marker (E‐cadherin and Claudin‐1) and consistently decreased migratory and invasive ability in CRC cells, while overexpression of HSPA8 resulted in the opposite phenotype (Figure 2C–L and Figure S4B–H, Supporting Information). In addition, HSPA8 depletion decreased the migratory ability and colony formation of RKO cells under Dabrafenib or Encorafenib (BRAF V600E inhibitors) treatment, indicating that HSPA8 plays a pivotal role in the drug response to BRAF inhibitors (Figure 2M–S). Together, these data show that HSPA8 promotes EMT in BRAF V600E CRC cells.

### 2.3 HSPA8 Activates the Wnt/β‐Catenin Pathway through CMA‐Mediated CAV1 Degradation

**Figure 3:**
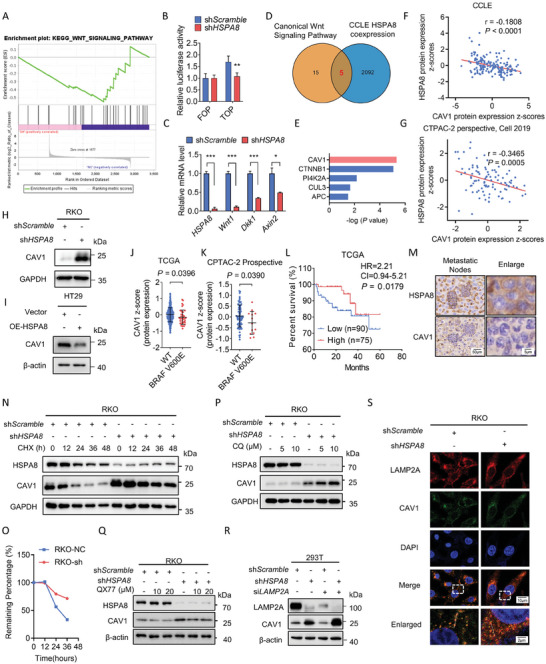
HSPA8 activates Wnt/β‐catenin pathway though CMA‐mediated CAV1 degradation. A) GSEA of the KEGG Wnt signaling pathway was performed in the RKO WT and RKO shHSPA8 groups. B) The relative luciferase activity in control cells transfected with FOP‐flash and TOP‐flash vectors and cells transfected with shscramble and shHSPA8 is shown. C) Real‐time qPCR analysis was performed to examine the mRNA expression levels (mean ± SEM) of canonical Wnt/β‐catenin signaling components. D) Venn diagram showing that five genes enriched in the canonical Wnt signaling pathway gene set and CCLE HSPA8‐related gene set. E) Validation of the correlation between candidate genes and HSPA8. F,G) HSPA8 expression levels showed a negative correlation with CAV1 expression levels according to the CCLE and cBioportal dataset CTPAC‐2 perspective (Pearson correlation test). H) Immunoblotting analysis of CAV1 expression in RKO cells stably expressing shScramble or shHSPA8. I) Immunoblotting analysis of CAV1 expression in HT29 cells stably expressing Vector or OE‐HSPA8. J,K) CAV1 mRNA levels in patients with BRAF WT or BRAF V600E mutation in TCGA or CTPAC‐2 perspective datasets (Student's *t* test). L) Overall survival of CRC patients according to TCGA dataset (*P* = 0.0179, log‐rank [Mantel–Cox] test). M) Representative images of HSPA8 and CAV1 immunohistochemical staining in liver metastatic nodules of mouse tissues, scale bar: 50 µm (left), 5 µm (right). N,O) Immunoblotting analysis of RKO cells stably expressing shScramble or shHSPA8 (80% to 90% confluent) treated with 100 µg mL^−1^ CHX or solvent. Statistical quantification of CAV1 expression at different time points. P) Immunoblotting analysis of RKO cells stably expressing shScramble or shHSPA8 treated with 5 µm, 10 µm CQ or solvent. Q) Immunoblotting analysis of RKO cells stably expressing shScramble or shHSPA8 treated with 10/20 µm QX77 or solvent. R) Immunoblotting analysis of HEK293T cells stably expressing shScramble or shHSPA8 treated with siNC or siLAMP2A. S) Immunofluorescence assays display colocalization between CAV1 and LAMP2A with or without HSPA8 knockdown. Scale bar: 10 µm (top and middle), 2 µm (bottom). ***P < 0.001, **P < 0.01, and data are the mean ± SEM from at least three independent experiments.

Activation of the Wnt/β‐catenin pathway is present in 96% of CRC patients and considered an important biological cause of CRC progression and drug resistance.^[^ ^10^, ^22^ ^]^ Interestingly, downregulation of the Wnt/β‐catenin pathway was significantly observed in shHSPA8 cells (Figure 3A). Furthermore, FOP/TOP flash experiments proved that knocking down HSPA8 inhibited the expression of Wnt/β‐catenin pathway‐related genes, which was further verified by measuring the mRNA level of these genes (Figure 3B,C). To verify the essential regulators participating in HSPA8‐mediated regulation of the Wnt/β‐catenin pathway, we overlapped HSPA8 co‐expressed proteins in the CCLE database (2097 genes) with the canonical Wnt/β‐catenin signaling pathway gene set (20 proteins). As a result, five candidate proteins that may be regulated by HSPA8 (Figure 3D) were identified, among which CAV1 was the most enriched protein even over β‐catenin (Figure 3E). Intriguingly, the expression profile of CAV1 was negatively related to the expression of HSPA8 according to public datasets (Figure 3F,G). CAV1 was upregulated in HSPA8 knockdown cells and downregulated in HSPA8‐overexpressing cells (Figure 3H,I). In CRC tissue samples, CAV1 expression was downregulated compared to that in normal tissues (Figure S5A–D, Supporting Information). Consistently, the expression of CAV1 was lower in BRAF V600E patients and metastatic CRC (Figure 3J,K and Figure S5E,F, Supporting Information), which was associated with a worse survival rate (Figure 3L). These data indicated that the expression of HSPA8 and CAV1 was negatively correlated, which was further validated by the IHC staining of patient tissues and the orthotopic mouse model (Figure 3M and Figure S5G,H, Supporting Information). Given the slightly changed RNA level of CAV1 (Figure S5I,J, Supporting Information), the downregulation of CAV1 may be regulated at the protein level rather than the transcriptional level. Cycloheximide (CHX) time‐course assays revealed that stable knockdown of HSPA8 in RKO cells significantly reduced CAV‐1 degradation (Figure 3N,O). The proteasomal and lysosomal pathways are major cellular protein degradation pathways for mammalian cells.^[^ ^14b^ ^]^ Notably, shHSPA8‐mediated upregulation of CAV1 was not significantly influenced by MG132 (a proteasome inhibitor) treatment, indicating that HSPA8 promoted the degradation of CAV1 in a proteosome‐independent manner (Figure S5K, Supporting Information). To ascertain the role of lysosome‐associated degradation of CAV1, we used CQ (a lysosomal degradation pathway inhibitor) and found a concentration‐dependent increase of CAV1 (Figure 3P).^[^ ^23^ ^]^ However, shHSPA8‐mediated upregulation of CAV1 was not affected by 3‐MA (a macroautophagy inhibitor) treatment (Figure S6A, Supporting Information). In addition, no obvious colocalization of LC3 and CAV1 was observed by immunofluorescent assays (Figure S6B–D, Supporting Information), suggesting that the degradation progress may be independent of macroautophagy. Considering that HSPA8 is an important chaperone molecule in CMA, RKO cells with stable HSPA8 knockdown were treated with the CMA activator QX77, and the expression of CAV1 was recovered (Figure 3Q). Correspondingly, CAV1 further accumulated after knocking down LAMP2A, another regulator in the CMA process (Figure 3R). The colocalization of LAMP2A and CAV1 was further visualized through an immunofluorescent assay (Figure 3S and Figure S6E,F, Supporting Information). Intriguingly, knockdown of HSPA8 promoted the colocalization of LC3 and CAV1, indicating that CAV1 may be degraded through macroautophagy in the absence of HSPA8 (Figure S6B–D, Supporting Information). In summary, HSPA8 activates the Wnt/β‐catenin pathway through CMA‐mediated CAV1 degradation.

### 2.4 HSPA8 Interacts with CAV‐1 through the KIFSN Motif

**Figure 4:**
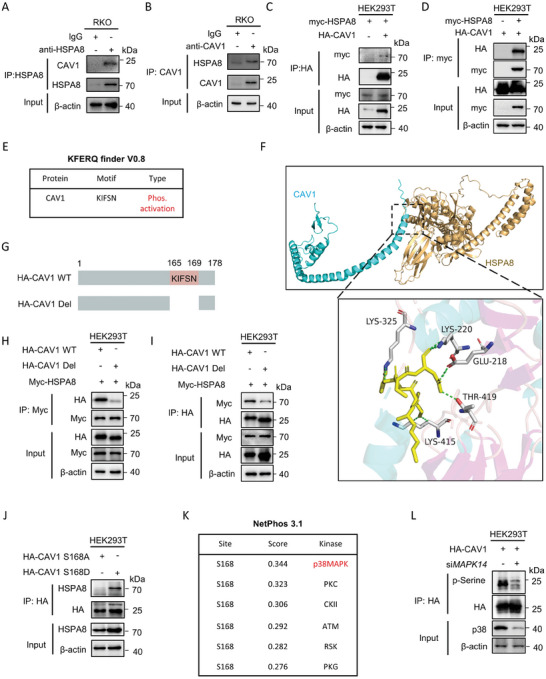
HSPA8 interacts with CAV‐1 through the KIFSN motif. A,B) The interaction between HSPA8 and CAV1 in RKO cells was determined by co‐IP assays. C,D) The interaction between myc‐HSPA8 and HA‐CAV1 in HEK293T cells was determined by co‐IP assays. E) KFERQ‐like motifs within the protein sequence of CAV1 were identified using KFERQ finder software v0.8. F) Molecular docking of 3D structures predicts the binding of the CAV1 KIFSN motif (yellow) with HSPA8. G) A schematic representation of HA‐CAV1 with full length (WT) and KIFSN motif deletion (Del). H,I) The interaction between myc‐HSPA8 and HA‐CAV1 (WT or Del) in HEK293T cells was determined by co‐IP assays. J) The interaction between HSPA8 and HA‐CAV1 with the S168A or S168D mutation in HEK293T cells was determined by co‐IP assays. K) Potential kinases to phosphorylate the CAV1 S168 site predicted by NetPhos 3.1. L) The interaction between HA‐CAV1 and p38 in HEK293T cells transfected with siNC or siMAPK14 was determined by co‐IP assays.

To further investigate the molecular mechanisms underlying HSPA8‐mediated CAV1 degradation, we examined the potential interaction between HSPA8 and CAV1. Co‐immunoprecipitation assays indicated the interaction between endogenous and exogenous HSPA8 and CAV1 (**Figure** **4**A–D). Considering that HSPA8 functions as an essential chaperone molecule by binding with client molecules of CMA, we examined whether HSPA8 bind with CAV1 through the HSPA8‐specific KFERQ‐like recognition motifs.^[^ ^12^, ^24^ ^]^ The sequence of CAV1 was analyzed through KFERQ finder V0.8,^[^ ^25^ ^]^ and revealed an evolutionarily conserved KFERQ‐like motif KIFSN (Figure 4E and Figure S7A, Supporting Information). We then performed docking analysis and found that CAV1 had a potential binding affinity with HSPA8 (Figure 4F and Figure S7B, Supporting Information). Deletion of the KIFSN motif abrogated the interaction between HSPA8 and CAV1 (Figure 4G–I). Consistent with the predicted results in Figure 4E, the binding site on HSPA8 contained positively charged amino acids, which could enhance its affinity for negatively charged S168‐phosphorylated CAV1. Compared to non‐phosphorylatable CAV1 S168A, CAV1 S168D mimicked phosphorylation and showed a remarkable interaction with HSPA8 (Figure 4J). Then, we used the NetPhos 3.1 server to predict the potential phosphokinase at S168 of CAV1, where p38 MAPK was highly scored (Figure 4K). We then validated the interaction between CAV1 and p38 MAPK through co‐immunoprecipitation assay (Figure S7C,D, Supporting Information). Furthermore, knocking down MAPK14, a subunit of p38 MAPK, significantly downregulated the P‐serine level of CAV1 (Figure 4L). Next, we analyzed the relationship between CAV1 S168 phosphorylation and HSPA8‐mediated EMT. Replenishment of WT HA‐CAV1 or HA‐CAV1 S168A downregulated the expression of epithelial markers and inhibited cell migration and invasion, while replenishment of HA‐CAV1 S168D did not show a similar effect (Figure S7E–H, Supporting Information). In summary, CAV1 interacts with HPSA8 through the KIFSN motif, where phosphorylated S168 regulated by p38 MAPK is an essential site for this interaction.

### 2.5 HSPA8/CAV1/β‐Catenin Axis Activates EMT in BRAF V600E Colorectal Cancer Cells

**Figure 5:**
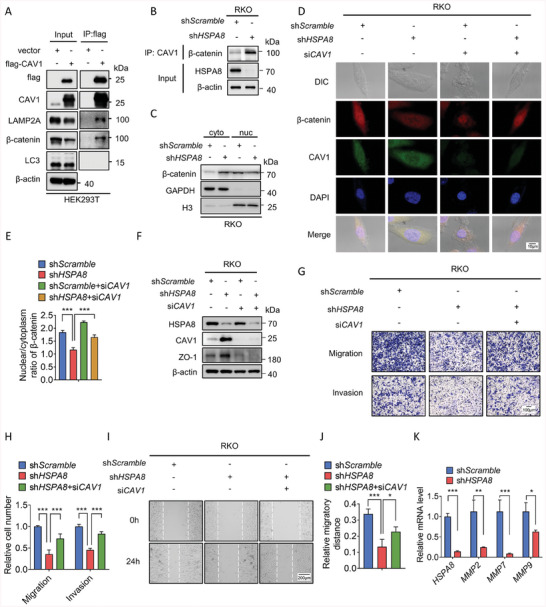
HSPA8 activates EMT by abrogating CAV1‐mediated inhibition of the β‐catenin/Wnt pathway in BRAF V600E CRC cells. A) The interactions between CAV1 and several proteins, including LAMP2A, β‐catenin, and LC3, in HEK293T cells were determined by co‐IP assays. B) The interaction between CAV1 and β‐catenin in RKO cells stably expressing shScramble or shHSPA8 was determined by co‐IP assays. C) Immunoblotting analysis of β‐catenin protein expression in the cytoplasm or nucleus in RKO cells stably expressing shScramble or shHSPA8. GAPDH is used as cytoplasmic marker and Histone 3 (H3) is a nuclear marker. D,E) Immunofluorescence assays display the subcellular localization of β‐catenin in RKO cells transfected with shScramble, shHSPA8, shScramble+siCAV1, or shHSPA8+siCAV1. Scale bar: 10 µm. F) Immunoblotting analysis of the expression of HSPA8, CAV1, and ZO‐1 in RKO cells transfected with shScramble, shHSPA8, shScramble+siCAV1, or shHSPA8+siCAV1. G,H) Transwell assays showing the cell migration and invasion of RKO cells transfected with shScramble, shHSPA8, or shHSPA8+siCAV1. Scale bar: 100 µm. I,J) Wound healing assay showing the migration of RKO cells transfected with shScramble, shHSPA8, or shHSPA8+siCAV1 after 24 h. Scale bar: 200 µm. K) Real‐time qPCR analysis was performed to examine the mRNA expression levels (mean ± SEM) of MMPs in RKO cells stably expressing shScramble or shHSPA8. ***P < 0.001, **P < 0.01, *P < 0.05, and data are the mean ± SEM from at least three independent experiments.

In light of the above findings, we hypothesized that the HSPA8/CAV1/β‐catenin axis might play a role during the EMT process in BRAF V600E CRC. Recent studies indicate that CAV1 can prevent the translocation of β‐catenin into the nucleus by trapping β‐catenin in the cytoplasm.^[^ ^17^, ^26^ ^]^ HSPA8‐mediated CAV1 degradation may release β‐catenin into the nucleus, leading to the activation of the Wnt/β‐catenin pathway and EMT. The interactions between CAV1, β‐catenin and LAMP2A were validated through co‐immunoprecipitation (**Figure** **5**A). HSPA8 depletion increased the interaction between CAV1 and β‐catenin (Figure 5B), leading to reduced nuclear β‐catenin (Figure 5C–E). In shHSPA8 cells, the epithelial marker ZO‐1 was increased compared to shScramble cells, which could be reversed by CAV1 knockdown (Figure 5F). Besides, impaired migratory, invasive, wound healing ability of CRC cells and β‐catenin nuclear translocation caused by HSPA8 depletion could be restored through knockdown of CAV1 (Figure 5G–J and Figure S8, Supporting Information). Consistently, overexpression of CAV1 decreased the nuclear translocation of β‐catenin and the migratory potential of CRC cells, indicating the pivotal role of CAV1 in CRC progression (Figure S9, Supporting Information). As a result, the expression of MMP2/7/9 was also affected by the depletion of HSPA8 (Figure 5K). These results indicate that HSPA8 activates EMT by abrogating CAV1‐dependent inhibition of the Wnt/β‐catenin pathway.

### 2.6 HSPA8 Inhibitor VER155008 Showed a Synergistic Effect with BRAF Inhibitors in BRAF V600E Colorectal Cancer Cells

**Figure 6:**
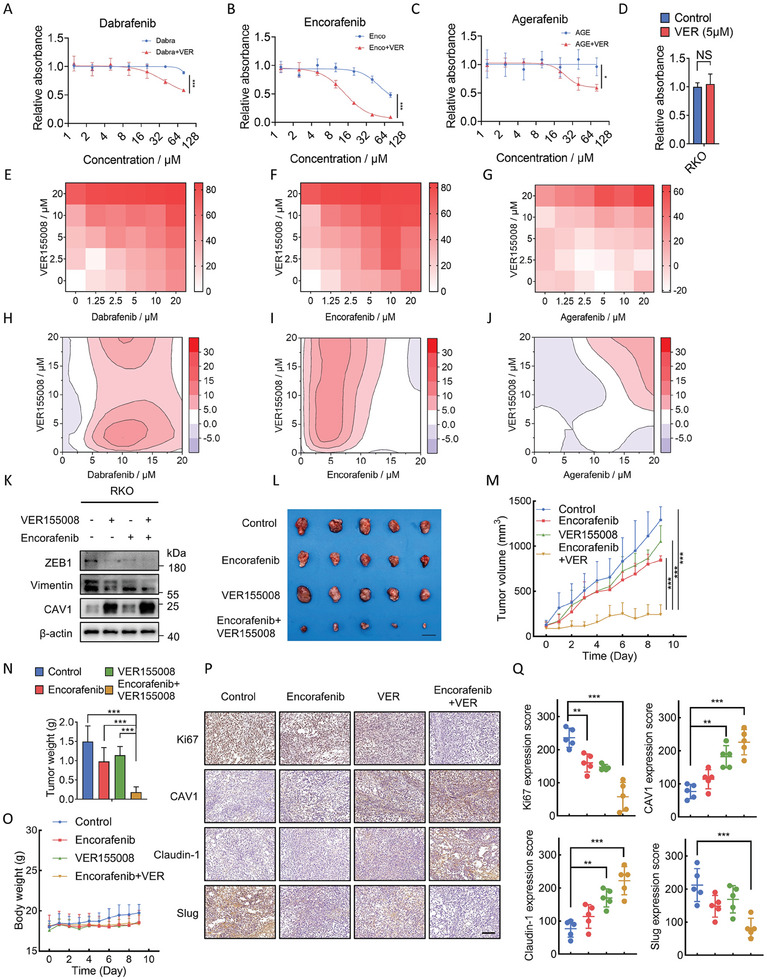
The HSPA8 inhibitor VER155008 showed a synergistic effect with the BRAF inhibitors in BRAF V600E CRC. A–D) Cell viability assay of RKO cells treated with VER155008 (5 µm), with the indicated concentration of Dabrafenib, Encorafenib, or Agerafenib for 24 h. E–J) The drug combination dose‐response matrices of VER155008 with Dabrafenib, Encorafenib, or Agerafenib in RKO cells. The drug interaction landscapes were calculated based on the ZIP model. K) Immunoblotting assays for HSPA8, CAV1, and EMT marker proteins levels in RKO cells treated with solvent, VER155008, Encorafenib, or VER155008+Encorafenib. L) Representative images of isolated tumors. Scale bar: 1 cm. M) The volume of tumors from each group (5 mice per group) was measured at the indicated time points. N) Tumor weight from each group (5 mice per group) was measured. O) The body weight of mice in each group was measured at the indicated time points. P,Q) Representative immunohistochemistry images of tumors treated with or without VER155008 or Encorafenib. The expression score of Ki67, CAV1, Claudin‐1, and Slug were calculated. Scale bar: 200 µm. ***P < 0.001, **P < 0.01, *P < 0.05, and data are the mean ± SEM from at least three independent experiments.

The critical role of HSPA8 in regulating BRAF V600E CRC prognosis prompted us to explore drug combination strategies involving BRAF V600E inhibitors and HSPA8 inhibitors. We first examined the IC_50_ of BRAF V600E inhibitors (Dabrafenib, Encorafenib, and Agerafenib) and the HSPA8 inhibitor VER155008.^[^ ^16^, ^27^ ^]^ As shown in **Figure** **6**A–D and Figure S10A–F (Supporting Information), separate use of both kinds of inhibitors showed a relatively higher IC_50_, while the combination of BRAF V600E inhibitors with VER155008 showed a significantly lower value of IC_50_. In addition, the Chou–Talalay method was used to calculate the combination index (CI), and the results showed synergistic effects of the combinational use of HSPA8 inhibitors with BRAF V600E inhibitors (Figure 6E–J and Figure S10G–L, Supporting Information). Moreover, a combination of VER155008 and Encorafenib had a more pronounced inhibitory effect on the EMT markers expression, cell migration, invasion, and wound healing (Figure 6K and Figure S11, Supporting Information). The combination of VER155008 and Encorafenib also showed a significant antitumor effect in the mouse model (Figure 6L–N). Moreover, no noticeable body weight loss or morphological changes in the main organs were observed (Figure 6O and Figure S10M, Supporting Information), indicating a low toxicity of this treatment in mice. Furthermore, the combination of VER155008 and Encorafenib recovered the expression of CAV1 and inhibited cell proliferation and EMT, as evidenced by repressed Ki‐67 and slug as well as enhanced CAV1 and Claudin‐1 by IHC staining (Figure 6P,Q). In summary, these data indicate the potent antitumor effect and satisfactory biosafety of combinational use of VER155008 and Encorafenib for potential clinical use in CRC treatment.

## 3 Discussion

The BRAF V600E mutation has been recognized as an important marker of worse prognosis for metastatic CRC patients. Recent studies have explored the underlying mechanisms of adaptive drug resistance in BRAF V600E melanoma.^[^ ^28^ ^]^ Activation of BRAF downstream pathways (MAPK cascade) or the PI3K‐Akt pathway may occur during BRAF V600E inhibition, leading to cancer refractoriness.^[^ ^29^ ^]^ These studies shed light on the mechanism underlying BRAF V600E cancer drug resistance, especially in melanoma. Although targeted drugs have been developed, the response rate may reach 80% in melanoma but just 5% in CRC, eliciting the urgent need for mechanistic research on the poor prognosis of BRAF V600E CRC patients. Several recent studies indicate that activation of the Wnt pathway may be responsible for the poor prognosis of CRC compared to other tumors (e.g., melanoma), but research on the underlying molecular mechanisms is still scarce. In this study, we identified HSPA8 as an essential biomarker that affected the prognosis of BRAF V600E CRC patients by modulating the degradation of CAV1 and translocation of β‐catenin to the nucleus. Specifically, HSPA8 recognizes CAV1 by KIFSN motif, leading to CMA‐dependent degradation of CAV1. Although the precise mechanism of CAV1 translocation from plasma membrane to lysosome needs further investigation, knockdown of HSPA8 or deletion of KIFSN motif can significantly impair the degradation of CAV1, indicating that HSPA8 and the interaction between HSPA8 and CAV1 are essential for CAV1 degradation. The first example of CMA‐mediated membrane protein degradation was EGFR. It has been reported that EGFR might be translocated to the cytoplasm by the ER‐Golgi transport process before localization on the plasma membrane or during receptor recycling.^[^ ^30^ ^]^ Similarly, RyR2, another integral membrane protein, is also degraded via CMA.^[^ ^31^ ^]^ These examples suggested that HSPA8 may interact and transfer CAV1 when CAV1 is on the endosomes or other structures, which needs further investigation.

HSPA8 has been recognized as a new biomarker for tumor progression in multiple human cancer types.^[^ ^32^ ^]^ However, the underlying mechanisms are largely unknown. HSPA8 can act as a molecular chaperone to assist in the correct folding of unfolded polypeptides and promote the selective degradation of various proteins through the CMA pathway, which is of great significance for maintaining cellular homeostasis. HSPA8 has been reported to play an essential role in metastasis in several types of cancers.^[^ ^33^ ^]^ For instance, HSPA8 promoted breast cancer metastasis by degrading Dicer in breast cancer cells.^[^ ^34^ ^]^ In addition, DJ‐1 bound to HSPA8 to promote Smad3 phosphorylation and nuclear aggregation in a protein‐interaction–dependent manner, thereby activating the TGF‐β pathway and esophageal squamous cell carcinoma metastasis.^[^ ^35^ ^]^ However, the role and underlying mechanisms of HSPA8‐mediated CRC metastasis are largely unknown. A peptide directly interacting with HSPA8 named P140 (also known as Lupuzor) has completed phase III evaluation (NCT01240694). In addition, 15‐deoxyspergualin is currently in a phase I/II clinical trial (NCT00709722), suggesting its safety and stability in clinical use. These achievements of HSPA8 inhibitors demonstrate the potential for clinical practice in CRC treatment. In this study, we found that high expression of HSPA8 is responsible for the persistent activation of the Wnt/β‐catenin pathway, which depends on HSPA8‐mediated degradation of CAV1. Compared to transcriptional modulation, regulation of protein degradation is rapid, enabling tumor cells to quickly cope with complex stresses, including targeted therapeutics. Apart from regulating the Wnt/β‐catenin pathway, HSPA8 may directly upregulate the MAPK cascade to promote CRC progression and desensitize cancer cells to BRAF V600E inhibitors, which is consistent with previous reports.^[^ ^36^ ^]^ Furthermore, HSPA8 inhibitors show synergistic effects with BRAF V600E inhibitors in CRC cells, indicating their potential for clinical use.

Aberrant activation of the Wnt/β‐catenin pathway is one of the most significant signatures of CRC, which may contribute to the different drug responses from other types of tumors. Recent studies have revealed that activation of the Wnt/β‐catenin pathway contributes to adaptive drug resistance to BRAF inhibitors in CRC.^[^ ^37^ ^]^ However, effective clinical strategies targeting the Wnt/β‐catenin pathway are currently unavailable probably due to the on‐target toxicity,^[^ ^38^ ^]^ which prompts the exploration of clinical strategy based on other targets. Intriguingly, CAV1 participates in regulating the Wnt/β‐catenin pathway by recruiting β‐catenin to caveolae membrane domains,^[^ ^17^, ^39^ ^]^ suggesting its essential role in regulating the Wnt/β‐catenin pathway. Due to its important role in tumorigenesis and tumor progression, CAV1 has attracted extensive attention in recent years.^[^ ^39^ ^]^ Loss of CAV1 is widely reported in the progression of various tumors, including CRC, and is closely related to tumor metastasis and drug resistance.^[^ ^40^ ^]^ Recent studies have also reported a potential link between CAV1, BRAF V600E mutation and the downstream signaling pathways of BRAF.^[^ ^41^ ^]^

In summary, our study sheds light on the role of HSPA8 in regulating the progression of BRAF V600E CRC through CMA‐dependent degradation of CAV1. Depleted CAV1 leads to the nuclear translocation of β‐catenin and subsequent activation of EMT process. Therefore, HSPA8 may serve as a predictive and prognostic biomarker for CRC patients, as well as a promising therapeutic target to relieve drug resistance in patients with BRAF V600E mutation.

## 4 Experimental Section

### Antibodies and Reagents

The following antibodies were used in this study: β‐actin (sc‐1616, Santa Cruz Biotechnology), LC3 (NB100‐2220, Novus, Saint Charles, MO, USA), HSPA8 (NB120‐2788, Novus, Saint Charles, MO, USA), CAV1 (3267, Cell Signaling Technology), E‐cadherin (3195, Cell Signaling Technology), ZO‐1 (8193, Cell Signaling Technology), ZEB1 (70 512, Cell Signaling Technology), vimentin (5741, Cell Signaling Technology), slug (9585, Cell Signaling Technology), snail (3879, Cell Signaling Technology), Claudin‐1 (13 255, Cell Signaling Technology), HA‐tag (ab49969, Abcam), LAMP2A (ab23322, Abcam), Flag‐tag (14 793, Cell Signaling Technology), Myc‐tag (2278, Cell Signaling Technology), Histone‐H3 (4499, Cell Signaling Technology), β‐catenin (8480, Cell Signaling Technology), BRAF (14 814, Cell Signaling Technology), p38 MAPK (8690, Cell Signaling Technology), P‐serine (530 893, ZEN‐Bioscience), GAPDH (250 133, ZEN‐Bioscience), and goat anti‐rabbit IgG (HRP) (sc‐2004; Santa Cruz Biotechnology). In immunofluorescent assays, anti‐rabbit Alexa Fluor 488 (A27012), anti‐mouse Alexa Fluor 488 (A10667), anti‐rabbit Alexa Fluor 594 (A32740), and anti‐mouse Alexa Fluor 594 (A11005) were purchased from Thermo Fisher Scientific.

The following reagents were used in this study: Agerafenib (HY‐15200), 3‐methyladenine (3‐MA) (HY‐19312), chloroquine (HY‐17589A), MG132 (HY‐13259), bafilomycin A1 (Baf A1; HY100558), and QX77 (HY‐112483), which were purchased from Med Chem Express. MTT (M2128), DMSO (D2650), chloroquine diphosphate salt (C6628), and crystal violet (C0775), were obtained from Millipore Sigma. Dabrafenib (SD5919) was purchased from Beyotime Biotechnology. Encorafenib (T6487) was obtained from TargetMol, USA. VER155008 (S7751) and CHX (S7418) were purchased from Selleck.

### Cell Culture

The human BRAF V600E CRC cell lines RKO, HT29, and other CRC cell lines HCT116, SW480, SW48, SW620, and LOVO were purchased from the ATCC (Manassas, VA, USA) and maintained in DMEM (C11995500BT, Gibco) or RPMI1640 (C11875500BT, Gibco) supplemented with 10% fetal bovine serum (10 100 147, Gibco), 100 U mL^−1^ penicillin/streptomycin (C0222, Beyotime Biotechnology) in incubators at 37 °C under 5% CO_2_ atmosphere. To generate HSPA8 stable knockdown and overexpression cells, lentivirus harboring shRNA (5′‐ATATGAAACATTGGCCCTTTA‐3′) or cDNA for HSPA8 was used to infect CRC cells, followed by puromycin selection.

### Animal Model

Animal experiments were approved by the Institutional Animal Care and Treatment Committee of Sichuan University (20 221 214 002). 6–8 week‐BALB/c nude mice with equal numbers of female and male were obtained from GemPharmatech and randomly assigned. Mice in experimental groups were provided mouse chow and sterile water in pathogen‐free microisolators. For the subcutaneous CRC xenograft model, 100 µL PBS suspended 1 × 10^7^ RKO and was injected subcutaneously into the flanks of mice, as previously described.^[^ ^42^ ^]^ Mice were randomly grouped when the volume of each tumor reached ≈100 mm^3^ and was administered 100 µL of vehicle, Encorafenib (3 mg kg^−1^ in physiological saline, administered intraperitoneally every 2 days) or VER155008 (3 mg kg^−1^ in physiological saline, administered intraperitoneally every 2 days). The tumor volumes were measured using calipers every day in a blinded manner and calculated according to the formula: tumor volume (mm^3^) = (length × width^2^)/2.

### Detection of Cell Growth

The MTT assay was used to detect the cytotoxic effect of agents on tumor cell viability, and the details were previously described.^[^ ^43^ ^]^ Briefly, cells (5 × 10^3^ cells per well) were seeded in 96‐well plates and treated with indicated agents for 24 h. The absorbance was measured at 570 nm wavelength with a multiwell spectrophotometer.

The effect of agents on cell proliferation was analyzed by colony formation assay, as previously described.^[^ ^45^ ^]^ Cells (300 cells per well) were seeded in 24‐well plates and treated with indicated agents. Culture medium was changed every 2 days. The colonies were stained with crystal violet staining solution (C0121, Beyotime Biotechnology) for 2 h after 2 weeks.

### Immunoblotting and Immunoprecipitation

Cultured cells were lysed with RIPA buffer (1.0 mm EDTA, 50 mm Tris, 150 mm NaCl, 1% Triton X‐100, 0.1% SDS, 1 mm PMSF, 1% sodium deoxycholate). In co‐immunoprecipitation assays, cultured cells were lysed with IP lysis buffer (137 mm NaCl, 20 mm Tris, 10% glycerol, 2 mm EDTA, and 1% NP‐40, adjust to pH 8). The whole‐cell lysates were added with 1 mg of the indicated antibodies, then subjected to immunoprecipitation overnight at 4 °C. The immunoprecipitated protein was pulled down with protein A+G agarose (P2055, Beyotime Biotechnology) for 4 h. The samples were then analyzed by immunoblotting.

### Quantitative RT‐PCR

Total RNA was extracted using TRIzol reagent (15 596 018, Thermo Fisher Scientific), as previously described. ^[^ ^44^ ^]^ Reverse transcription was conducted using the PrimeScript RT reagent Kit with gDNA Eraser (RR047A, Takara). Primers and SYBR Green (FP205‐02, TIANGEN) were used to quantify the expression levels of the indicated mRNA. The real‐time PCRs were performed in triplicate on a CFX connect real‐time PCR detection system (Bio‐Rad).

### Immunofluorescence

Cells (5 × 10^3^ cells per well) were plated on glass coverslips for 24 h and fixed in 4% paraformaldehyde. 0.4% Triton X‐100 was used to permeabilize cultured cells and 5% fetal bovine serum was used for blocking. The indicated primary antibodies and Alexa Fluor secondary antibodies were incubated. Images were viewed with Carl Zeiss LSM 880.

### RNA Interference

CAV1, LAMP2A, and siRNA were synthesized by Gene Pharma. The siRNA sequences were as follows: human CAV1 siRNA #1, 5′‐AGACGAGCUGAGCGAGAAGCA‐3′; siRNA #2, 5′‐CAUCUACAAGCCCAACAACTT‐3′; LAMP2A siRNA: 5′‐GCGGUCUUAUGCAUUGG AATT‐3′; BRAF siRNA: 5′‐AGAACACTTGTGTGGTTAAAG‐3′. According to the manufacturer's protocol, the siRNA was transfected with Lipofectamine 3000 reagent (L3000015, Invitrogen) for 48 h.

### Immunohistochemistry

Xenograft tumors were fixed with 4% paraformaldehyde, embedded with paraffin, and sectioned on pre‐adherent slides. Slides were treated with indicated primary antibodies (1:200) overnight. Then, the sections were incubated with a secondary antibody and developed with 3,3′‐diaminobenzidine chromogen. The images of samples were captured with DM2500 fluorescence microscope (Danaher, Wetzlar, Germany). Quantitative scoring analyses were performed by multiplying the percentage of staining‐positive cells (A) by the intensity (B: 0, negative; 1, weakly staining; 2, moderate staining; 3, strong staining). The final score for each slide was calculated as the sum of A × B.

### Statistical Analysis

All statistical analyses and graphics were performed using GraphPad 9 software (GraphPad, La Jolla, CA, USA). Student's *t* test, one‐way ANOVA, or two‐way ANOVA were used to analyze statistical significance. All data are presented as the means ± SEM from at least three individual experiments. *P* < 0.05 was considered statistically significant.

## Conflict of Interest

The authors declare no conflict of interest.

## Supporting information

Supporting Information

## Data Availability

The data that support the findings of this study are available in the supplementary material of this article.
