# Supplementary material for: HSPA8 Activates Wnt/β‐Catenin Signaling to Facilitate BRAF V600E Colorectal Cancer Progression by CMA‐Mediated CAV1 Degradation
Source: Adv Sci (Weinh). 2023 Nov 16;11(3):2306535. doi: 10.1002/advs.202306535 (PMC10797426; doi:10.1002/advs.202306535)
Supplement: Supplementary file 1 — Supporting Information [file ADVS-11-2306535-s001.pdf]

ADVANCED SCIENCE
Open Access

Supporting Information

for *Adv. Sci.*, DOI 10.1002/advs.202306535

HSPA8 Activates Wnt/β-Catenin Signaling to Facilitate BRAF V600E Colorectal Cancer Progression by CMA-Mediated CAV1 Degradation

Bowen Li, Hui Ming, Siyuan Qin, Li Zhou, Zhao Huang, Ping Jin, Liyuan Peng, Maochao Luo, Tingting Zhang, Kui Wang, Rui Liu, Yih-Cherng Liou, Edouard C. Nice, Jingwen Jiang and Canhua Huang**

Supporting information

HSPA8 activates Wnt/β-catenin signaling to facilitate BRAF V600E colorectal cancer progression by CMA-mediated CAV1 degradation

Bowen Li [a,1], Hui Ming [a,1], Siyuan Qin [a,1], Li Zhou [a], Zhao Huang [a], Ping Jin [a], Liyuan Peng [a], Maochao Luo [a], Tingting Zhang [a], Kui Wang [a], Rui Liu [b], Yih-Cherng Liou [c,d], Edouard C. Nice [e], Jingwen Jiang [f,#], Canhua Huang [a,#].

[a] State Key Laboratory of Biotherapy and Cancer Center, West China Hospital and West China School of Basic Medical Sciences and Forensic Medicine, Sichuan University and Collaborative Innovation Center for Biotherapy, Chengdu, 610041, P.R. China.

[b] State Key Laboratory of Oral Diseases, National Clinical Research Center for Oral Diseases, Chinese Academy of Medical Sciences Research Unit of Oral Carcinogenesis and Management, West China Hospital of Stomatology, Sichuan University, Chengdu, Sichuan 610041, China.

[c] Department of Biological Sciences, Faculty of Science, National University of Singapore, Singapore 117543, Singapore.

[d] Graduate School for Integrative Sciences and Engineering, National University of Singapore, Singapore 117573, Singapore.

[e] Department of Biochemistry and Molecular Biology, Monash University, Clayton, VIC 3800, Australia.

[f] West China School of Public Health and West China Fourth Hospital, Sichuan University, Chengdu, 610041, P.R. China.

[1]These authors contributed equally to this work.

[#]Corresponding authors

Email Addresses: jjwcn@foxmail.com (J. Jiang); hcanhua@scu.edu.cn (C. Huang).

Figure S1. BRAF V600E mutation induces elevated HSPA8 expression in human colorectal cancer.

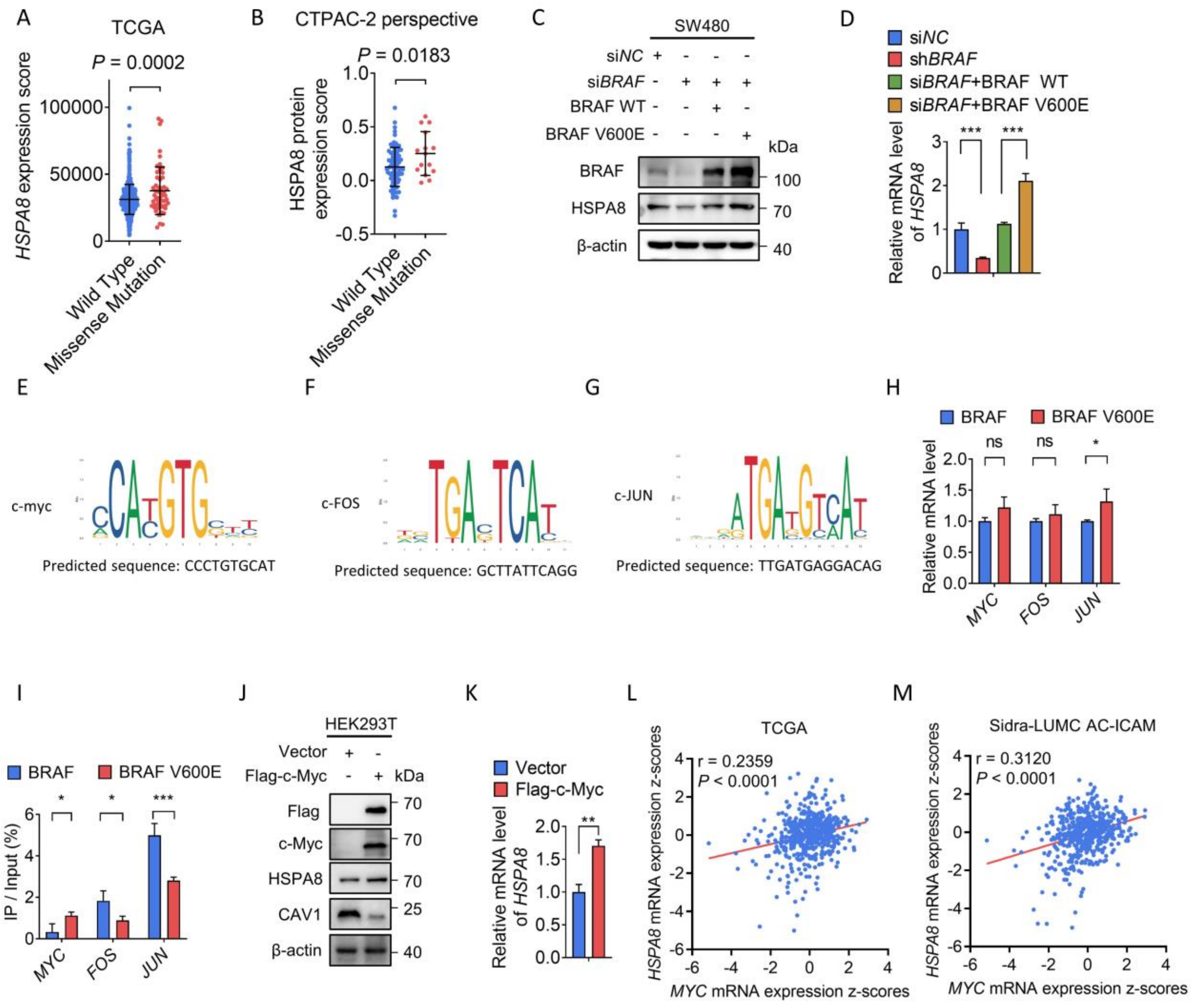

Figure S1. BRAF V600E mutation induces elevated HSPA8 expression in human colorectal cancer. (A-B) *HSPA8* mRNA levels in CRC patients with or without BRAF missense mutation according to TCGA and CTPAC-2 perspective dataset (Student's t test). (C) Immunoblotting assays of SW480 cells transfected with si*NC*, si*BRAF*, si*BRAF* + BRAF WT or si*BRAF* + BRAF V600E. (D) Real-time qPCR analysis was performed to examine the mRNA expression levels of *HSPA8* (mean ± SEM) in SW480 cells transfected with si*NC* or si*BRAF*, followed by overexpression of BRAF WT or BRAF V600E. (E-G) The predicted sequence of the interaction between BRAF downstream transcription factors (e.g., c-Myc, c-FOS, c-JUN) and the HSPA8 promoter according to JASPAR. (H) Real-time qPCR analysis was performed to examine the mRNA expression levels of *MYC*, *FOS*, or *JUN* (mean ± SEM) in SW480 cells transfected with BRAF WT or BRAF V600E plasmid. (I) ChIP-qPCR analysis. Chloroplasts fixed with formaldehyde (FA) were extracted from SW480 cells transfected with BRAF or BRAF V600E plasmid. Then, the DNA was sonicated into short fragments under specific conditions, immunoprecipitated with the indicated antibodies, and analyzed by quantitative real-time RT-PCR. Error bars indicate standard deviations for triplicates. (J) Immunoblotting assays of HSPA8 expression in HEK293T cells transfected with Vector or Flag-c-Myc plasmid. (K) Real-time qPCR analysis was performed to examine the *HSPA8* mRNA expression levels (mean ± SEM) in HEK293T cells transfected with Vector or Flag-c-Myc plasmid. (L-M) HSPA8 expression levels showed a positive correlation with MYC expression levels according to the TCGA and cBioportal dataset Sidra-LUMC AC-ICAM (Pearson correlation test). ***$P < 0.001$, **$P < 0.01$, *$P < 0.05$, and data are the mean ± SEM from at least three independent experiments.

Figure S2. HSPA8 expression is positively correlated with metastasis in human colorectal cancer.

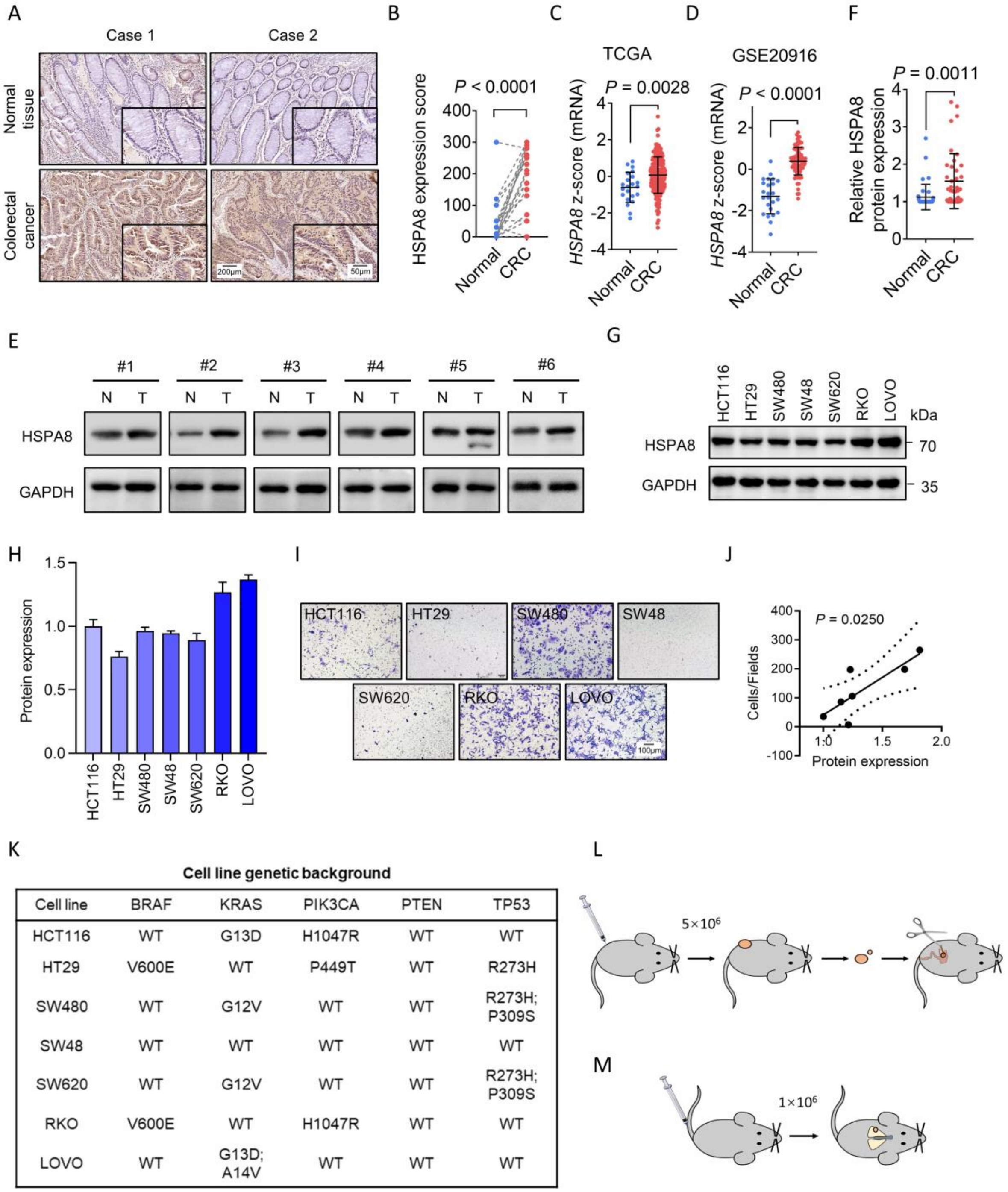

Cell line	BRAF	KRAS	PIK3CA	PTEN	TP53
HCT116	WT	G13D	H1047R	WT	WT
HT29	V600E	WT	P449T	WT	R273H
SW480	WT	G12V	WT	WT	R273H; P309S
SW48	WT	WT	WT	WT	WT
SW620	WT	G12V	WT	WT	R273H; P309S
RKO	V600E	WT	H1047R	WT	WT
LOVO	WT	G13D; A14V	WT	WT	WT

Figure S2. HSPA8 expression is positively correlated with metastasis in human colorectal cancer. (A) Representative images of HSPA8 immunohistochemical staining in normal colorectal tissue or CRC tissue. Scale bar: 200 μm, 50 μm (enlarged). (B) Statistical quantification of HSPA8 immunohistochemical staining in normal colorectal tissue or CRC tissue (P < 0.0001, paired Student's t test). (C-D) *HSPA8* mRNA levels in normal or CRC patients according to TCGA and GSE20916 dataset (P = 0.0028 and P < 0.0001, Student t test). (E-F) Immunoblotting analysis of HSPA8 expression levels in normal colorectal tissue or CRC tissue (P = 0.0011, Student's t test). (G) Immunoblot analysis of HSPA8 expression levels in several CRC cell lines. (H) Statistical quantification of HSPA8 expression levels in (G). (I) Representative images of migrated colorectal cells. Scale bar: 100 μm. (J) HSPA8 expression levels showed a positive correlation with migrated cell numbers (P = 0.0250, Pearson correlation test). (K) The genetic background of selected colorectal cancer cell lines. (L-M) The steps of constructing an orthotropic or tail vein injection mice model of CRC.

Figure S3. HSPA8 modulates the drug response to BRAF inhibitors in BRAF V600E CRC.

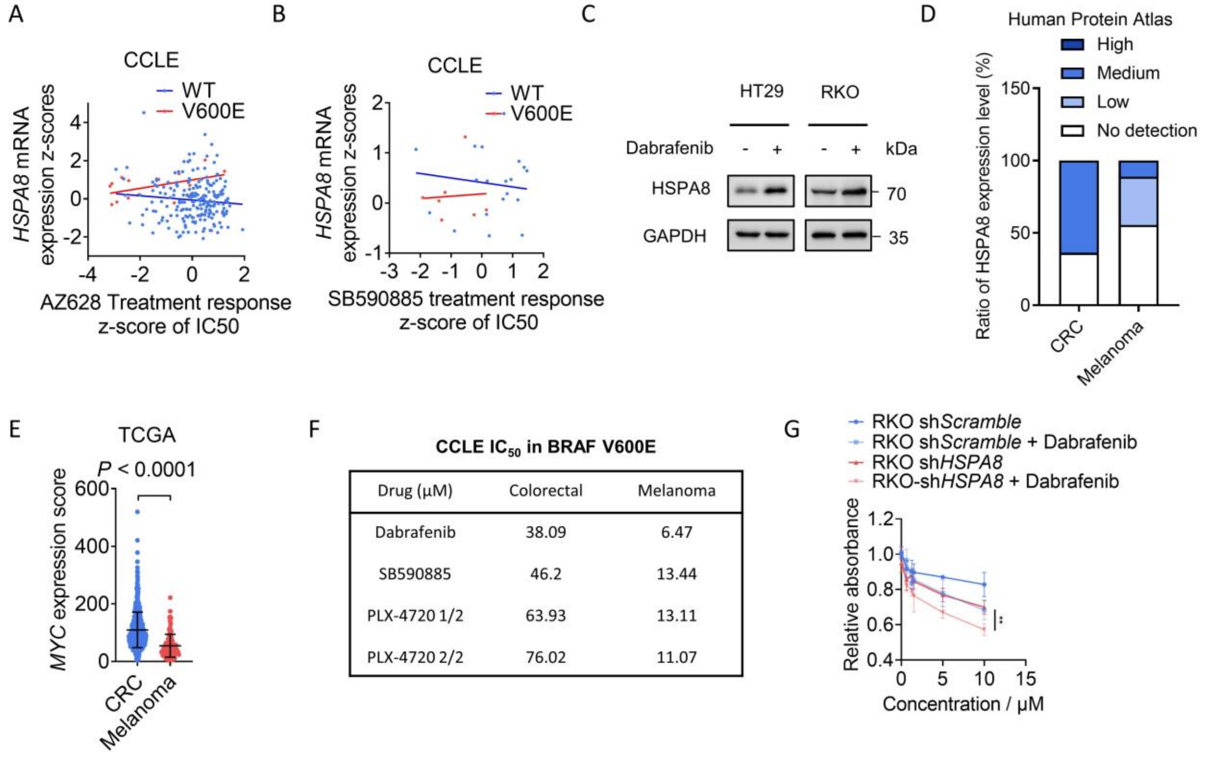

Drug (μM)	Colorectal	Melanoma
Dabrafenib	38.09	6.47
SB590885	46.2	13.44
PLX-4720 1/2	63.93	13.11
PLX-4720 2/2	76.02	11.07

Figure S3. HSPA8 modulates the drug response to BRAF inhibitors in BRAF V600E CRC. (A-B) *HSPA8* mRNA levels showed a different correlation with AZ628/SB590885 treatment response with or without BRAF V600E mutation, according to the CCLE database. (C) HT29 and RKO cell lines with the BRAF V600E genotype showed upregulated HSPA8 protein levels after dabrafenib treatment. (D) HSPA8 protein expression levels in CRC and melanoma tissues according to the Human Protein Atlas database. (E) *MYC* mRNA levels in CRC or melanoma patients according to TCGA dataset (Student's *t* test). (F) The IC50 values of the colorectal and melanoma BRAF V600E cell lines in the CCLE database were calculated. (G) Cell viability assay of RKO sh*Scramble* or sh*HSPA8* cells treated with or without the indicated concentrations of Dabrafenib for 24 h. ****P* < 0.001, ***P* < 0.01, **P* < 0.05, and data are the mean ± SEM from at least three independent experiments.

Figure S4. HSPA8 promotes epithelial-mesenchymal transition and metastasis in BRAF wild-type CRC cells.

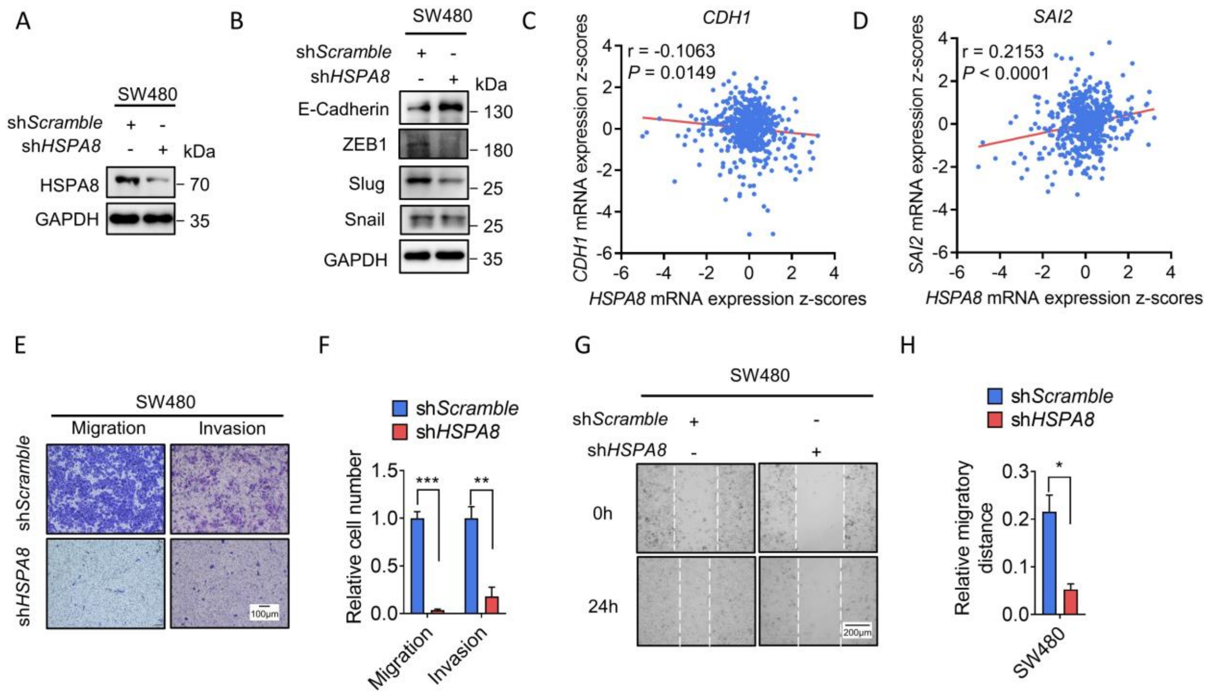

Figure S4. HSPA8 promotes epithelial-mesenchymal transition and metastasis in BRAF wild-type CRC cells. (A) Immunoblotting analysis of SW480 cells stably expressing sh*Scramble* or sh*HSPA8*. (B) Immunoblotting analysis of the effects of HSPA8 on the expression of EMT marker proteins in SW480 cells. (C) *HSPA8* mRNA levels showed a negative correlation with *CDH1* mRNA levels according to the cBioPortal dataset (P = 0.0149, Pearson correlation test). (D) *HSPA8* mRNA levels showed a positive correlation with *SAI2* mRNA levels according to the cBioPortal dataset ($P < 0.0001$, Pearson correlation test). (E-F) Effects of HSPA8 on cell migration and invasion evaluated by transwell assays in SW480 cells. Scale bar: 100 μm. (G-H) Wound healing assay showing the migration of SW480 cells stably expressing sh*Scramble* or sh*HSPA8* after 24 hours. Scale bar: 200 μm. ***$P < 0.001$, **$P < 0.01$, *$P < 0.05$. Values are the mean ± SEM from at least three independent experiments.

Figure S5. HSPA8 is negatively related with CAV1 expression in colorectal cancer.

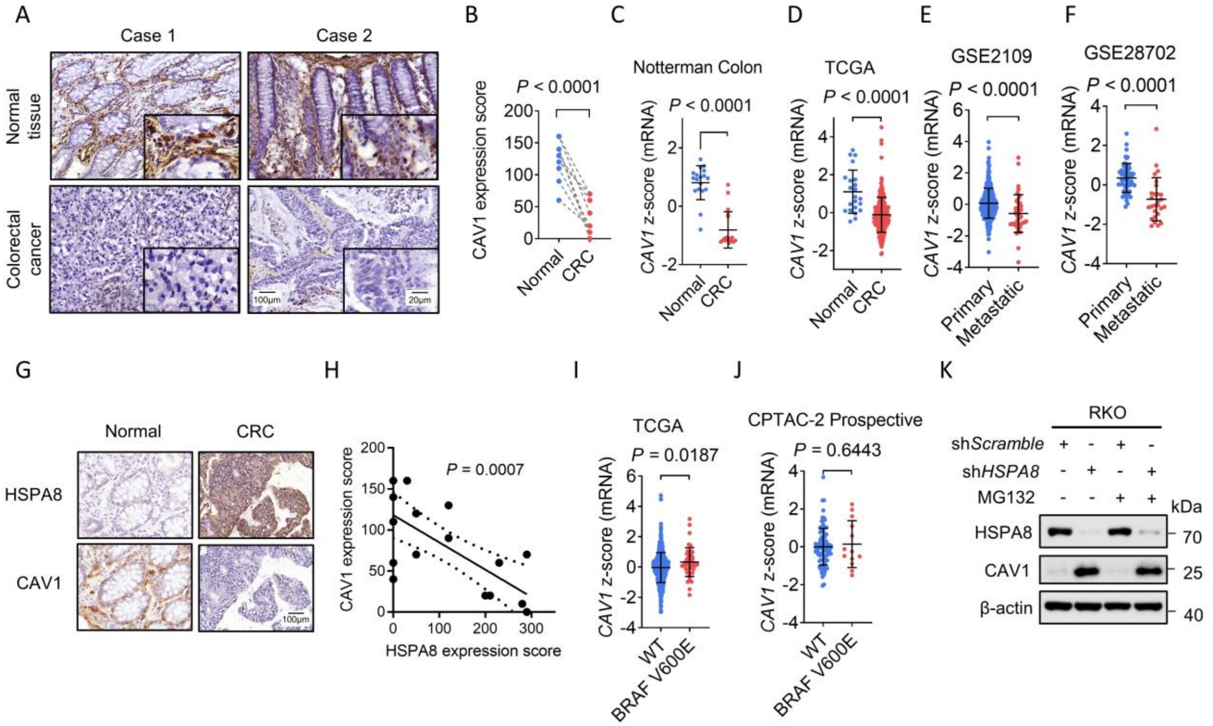

Figure S5. HSPA8 is negatively related with CAV1 expression in colorectal cancer. (A) Representative images of CAV1 immunohistochemical staining in normal colorectal tissue or CRC tissue. Scale bar: 100 μm, 20 μm (enlarged). (B) Statistical quantification of HSPA8 immunohistochemical staining in CRC tissues or normal tissues ($P < 0.0001$, paired Student's *t* test). (C-D) *CAV1* mRNA in CRC and adjacent tissues according to the Notherman Colon and TCGA databases. (E-F) CAV1 protein levels in primary and metastatic CRC patients according to GSE2109 and GSE28702 datasets (Student's *t* test). (G) Representative images of HSPA8 and CAV1 immunohistochemical staining in tissue samples. Scale bar: 100 μm. (H) HSPA8 expression score negatively correlated with CAV1 expression score ($P = 0.0007$, Pearson correlation test). (I-J) *CAV1* mRNA levels in CRC patients with or without BRAF V600E mutation according to TCGA and CPTAC-2 Prospective datasets (Student's *t* test). (K) Immunoblotting analysis of RKO cells stably expressing sh*Scramble* or sh*HSPA8* treated with or without 20 μM MG132 treatment.

Figure S6. HSPA8 downregulates CAV-1 through CMA instead of macroautophagy .

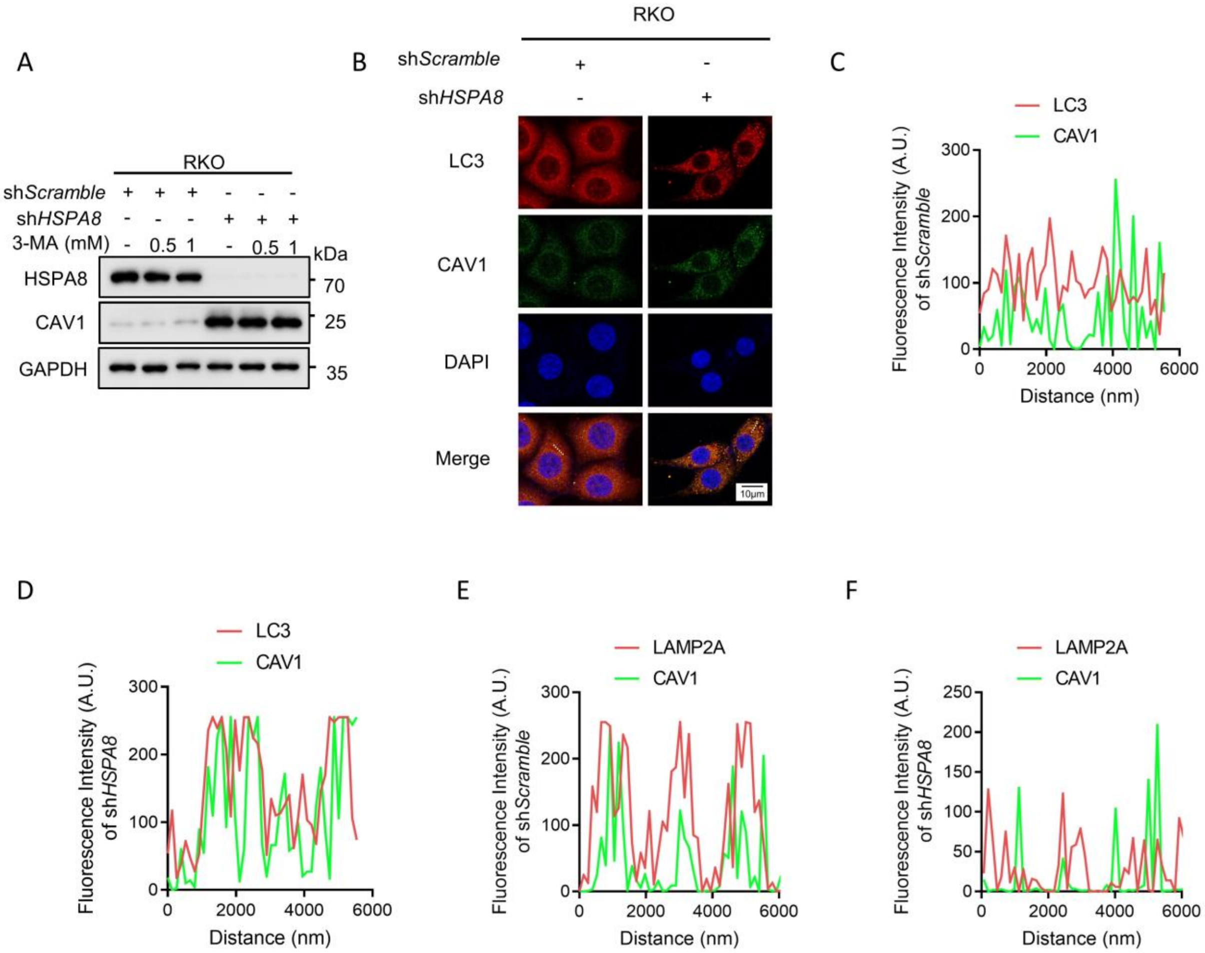

Figure S6. HSPA8 downregulates CAV-1 through CMA instead of macroautophagy . (A) Immunoblotting analysis of RKO cells stably expressing sh*Scramble* or sh*HSPA8* treated with or without 0.5 mM/1 mM 3-MA. (B-D) Immunofluorescence assays display colocalization between CAV1 and LC3 with or without HSPA8 knockdown. Scale bar: 10 μm. (E-F) Immunofluorescence intensity of CAV1 and LAMP2A with or without HSPA8 knockdown in Figure 3S.

Figure S7. The KIFSN motif is essential for HSPA8-induced epithelial-mesenchymal transition in BRAF V600E CRC cells.

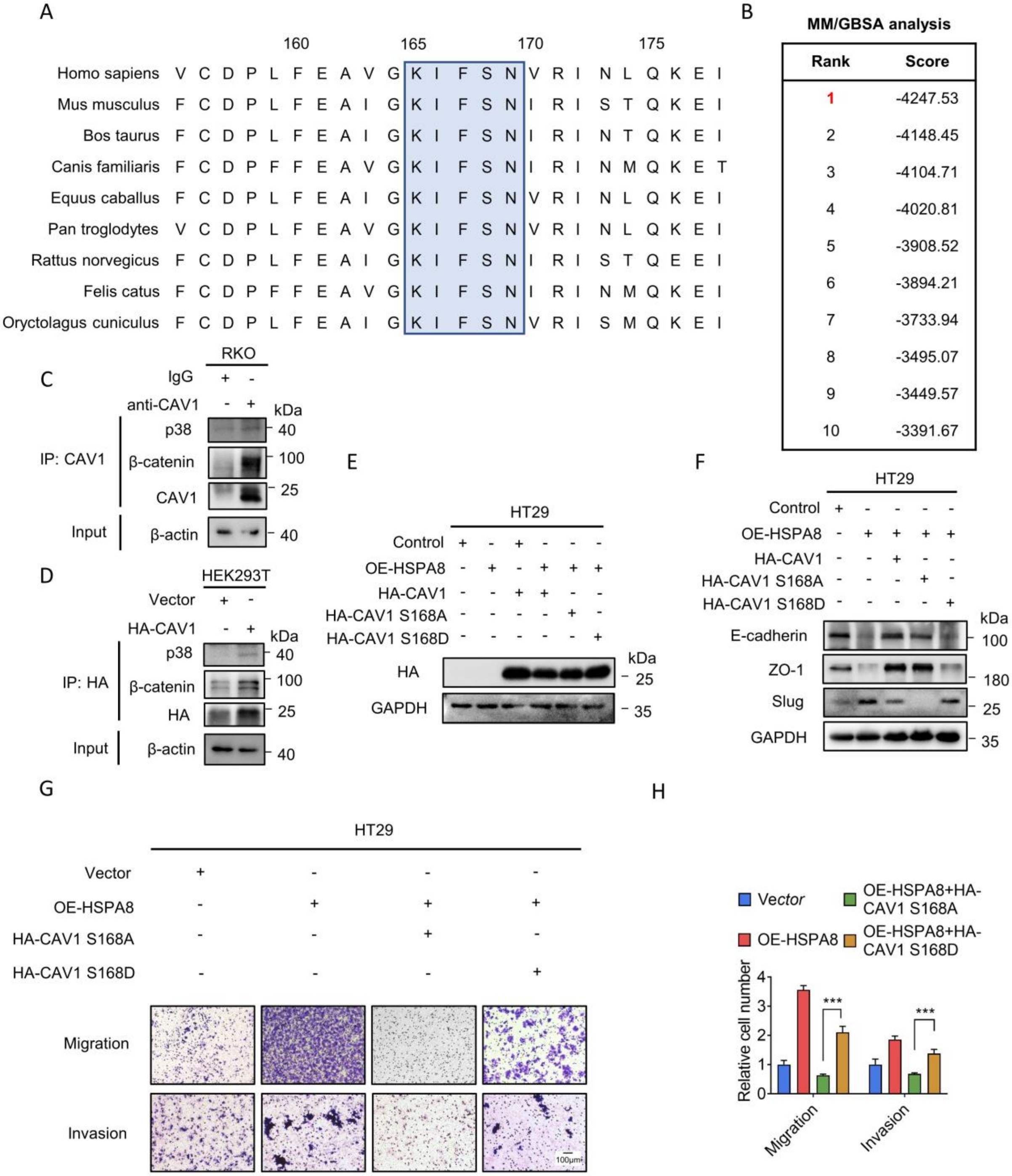

Figure S7. The KIFSN motif is essential for HSPA8-induced epithelial-mesenchymal transition in BRAF V600E CRC cells. (A) Default T-Coffee (Expresso) alignment of the CAV1 KIFSN motif. (B) Binding energy scores were calculated with MM-GBSA methods. (C-D) The interactions between CAV1 and p38, or CAV1 and β-catenin in RKO or HEK293T cells were determined by co-IP assays. (E-F) Immunoblotting assays for HA-tag or EMT marker proteins expression in HT29 cells transfected with Vector, OE-HSPA8, HA-CAV1, OE-HSPA8 + HA-CAV1, OE-HSPA8 + HA-CAV1 S168A, or OE-HSPA8 + HA-CAV1 S168D. (G-H) Transwell assays were conducted to examine the migratory and invasive ability of HT29 cells transfected with Vector, OE-HSPA8, OE-HSPA8 + HA-CAV1 S168A, or OE-HSPA8 + HA-CAV1 S168D. Scale bar: 100 μm. ***P < 0.001, and data are the mean ± SEM from at least three independent experiments.

Figure S8. HSPA8 activates the β-catenin/Wnt pathway by downregulating CAV-1.

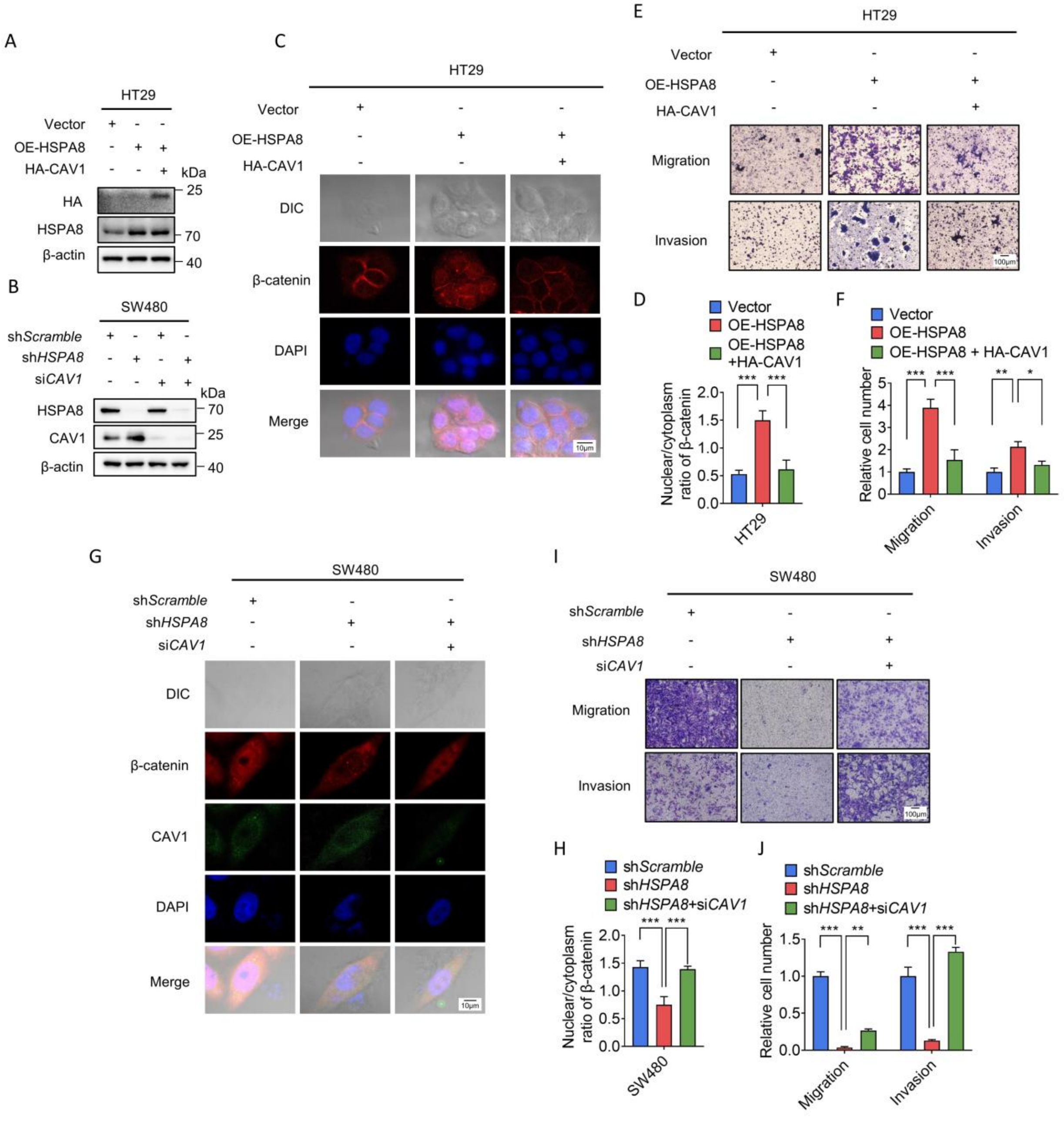

Figure S8. HSPA8 activates the β-catenin/Wnt pathway by downregulating CAV-1. (A) Immunoblotting assays of HSPA8, HA tag expression levels of HT29 cells transfected with Vector, OE-HSPA8, or OE-HSPA8 + HA-CAV1. (B) Immunoblotting assays of HSPA8, CAV1 expression levels in SW480 cells transfected with sh*Scramble* +si*NC*, sh*HSPA8* + si*NC*, sh*Scramble* + si*CAV1*, or sh*HSPA8* + si*CAV1*. (C-D) Immunofluorescence assays display the subcellular localization of β-catenin in HT29 cells transfected with Vector, OE-HSPA8, or OE-HSPA8 + HA-CAV1. Scale bar: 10 μm. (E-F) Transwell assays showing cell migration and invasion ability of HT29 cells transfected with Vector, OE-HSPA8, or OE-HSPA8 + HA-CAV1. Scale bar: 100 μm. (G-H) Immunofluorescence assays displaying the subcellular localization of β-catenin in SW480 cells transfected with sh*Scramble*, sh*HSPA8*, or sh*HSPA8* + si*CAV1*. Scale bar: 10 μm. (I-J) Transwell assays showing cell migration and invasion of SW480 cells transfected with sh*Scramble*, sh*HSPA8*, or sh*HSPA8* + si*CAV1*. Scale bar: 100 μm. ***$P < 0.001$, **$P < 0.01$, *$P < 0.05$, and data are the mean ± SEM from at least three independent experiments.

Figure S9. CAV1 inhibits β-catenin nuclear translocation and CRC migratory potential.

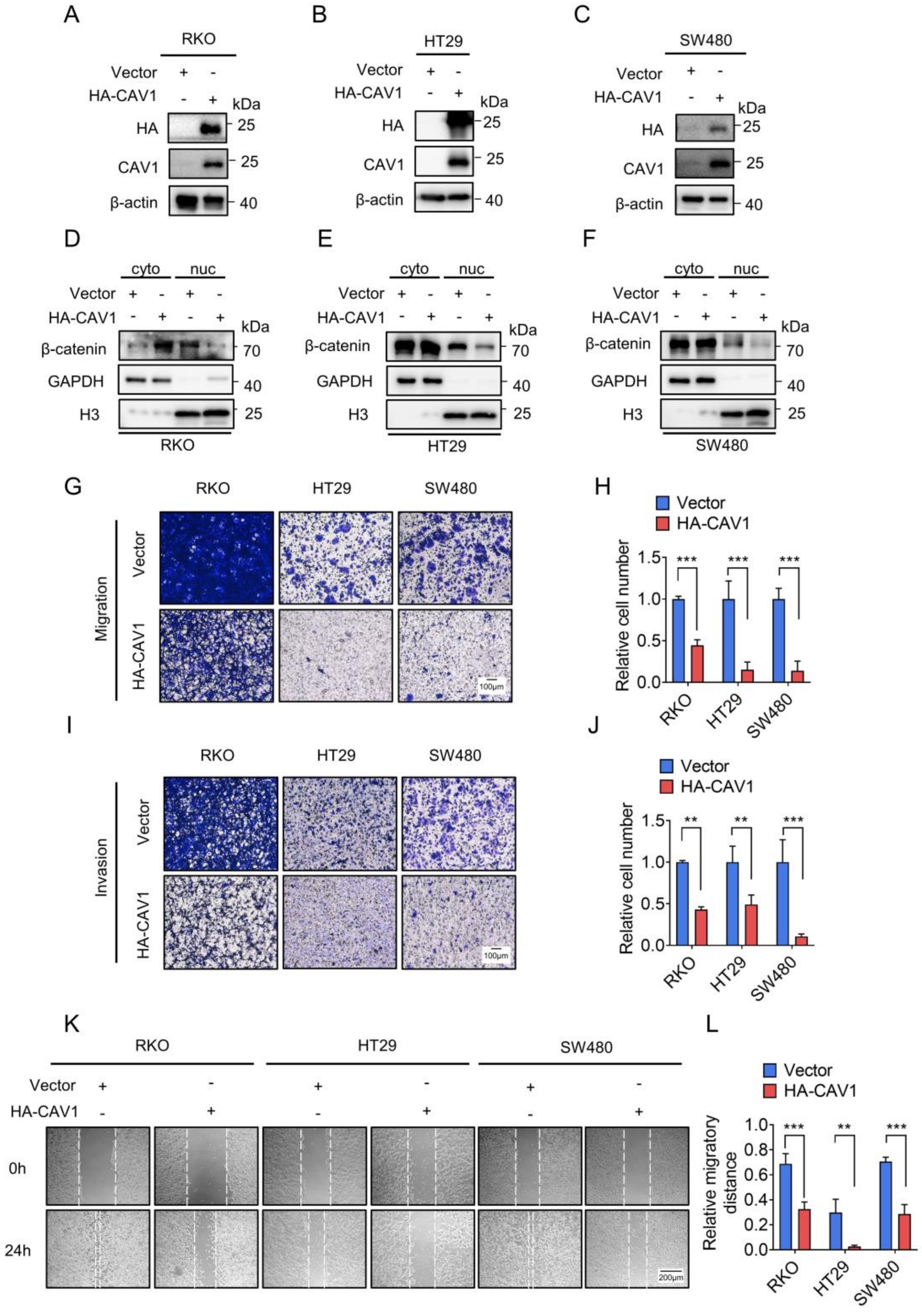

Figure S9. CAV1 inhibits β-catenin nuclear translocation and CRC migratory potential. (A-C) Immunoblotting analysis of RKO, SW480, HT29 cells transfected with Vector or HA-CAV1. (D-F) Immunoblotting analysis of the nuclear translocation of β-catenin in RKO, SW480, HT29 cells transfected with Vector or HA-CAV1. (G-J) Transwell assays showing the effects of CAV1 on cell migration and invasion in RKO, HT29, and SW480 cells transfected with Vector or HA-CAV1. Scale bar: 100 μm. (K-L) Wound healing assays showing the migration of RKO, HT29, and SW480 cells transfected with Vector or HA-CAV1 after 24 hours. Scale bar: 200 μm. ****P* < 0.001, ***P* < 0.01, **P* < 0.05, and data are the mean ± SEM from at least three independent experiments.

Figure S10. HSPA8 inhibitor VER155008 exhibits synergic effect with BRAF inhibitors on BRAF V600E HT29 cells.

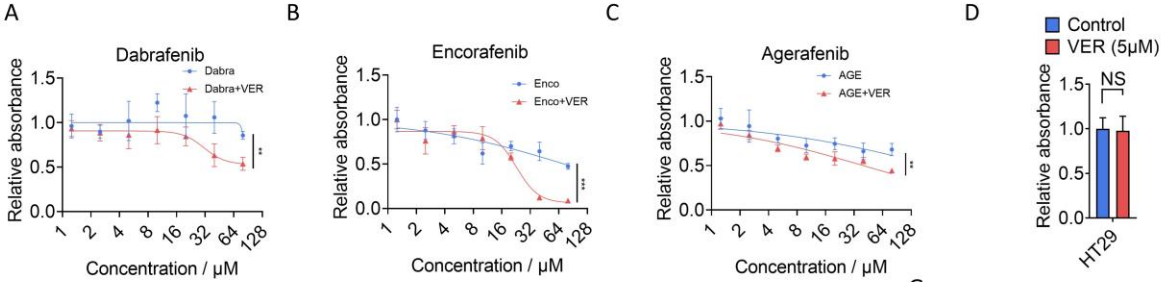

E

IC_{50}

Cell line	Drug	IC_{50}/μM
RKO	Dabrafenib	>100
RKO	Dabrafenib+VER	37.75
RKO	Encorafenib	51.28
RKO	Encorafenib+VER	14.09
RKO	Agerafenib	>100
RKO	Agerafenib+VER	24.24
RKO	VER155008	53.79

F

IC_{50}

Cell line	Drug	IC_{50}/μM
HT29	Dabrafenib	86.19
HT29	Dabrafenib+VER	31.03
HT29	Encorafenib	73.30
HT29	Encorafenib+VER	22.87
HT29	Agerafenib	>100
HT29	Agerafenib+VER	39.94
HT29	VER155008	37.58

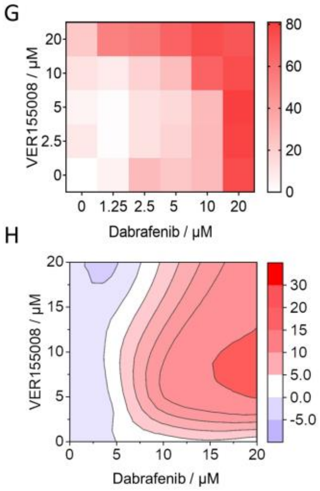

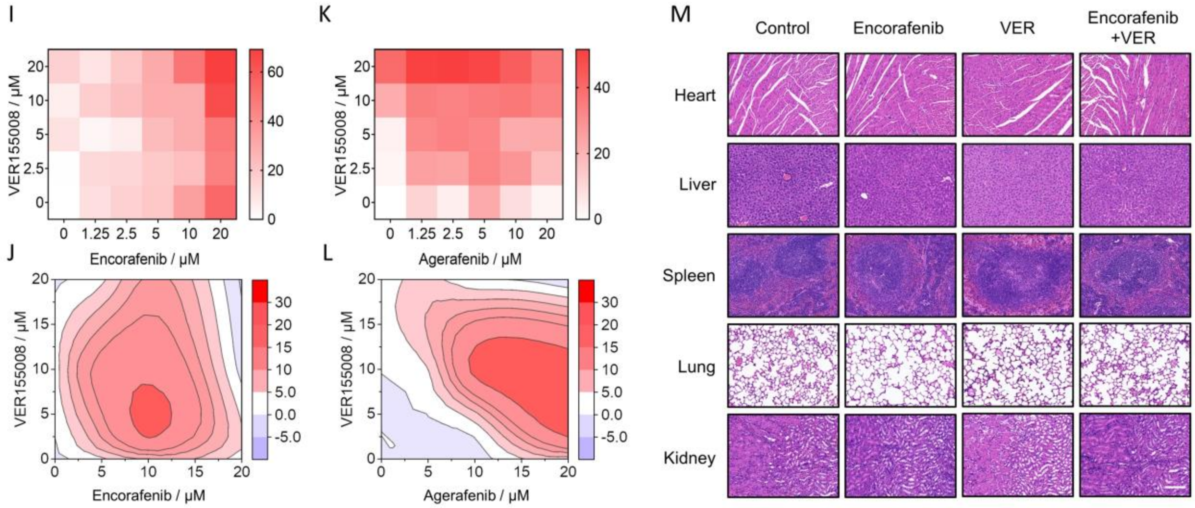

Figure S10. HSPA8 inhibitor VER155008 exhibits synergic effect with BRAF inhibitors on BRAF V600E HT29 cells. (A-D) Cell viability assay of HT29 cells treated with VER155008 (5 μM), with or without Dabrafenib, Encorafenib, or Agerafenib for 24 h. (E-F) Summary of the IC_{50} values of RKO and HT29 cells under the indicated treatments. (G-L) The drug combination dose-response matrices of VER155008 with Dabrafenib, Encorafenib, or Agerafenib in HT29 cells. The drug interaction landscapes were calculated based on the ZIP model. (M) Representative H&E images of the heart, liver, spleen, lung, and kidney of mice treated with or without VER155008, combined with or without Encorafenib. Scale bar: 200 μm. ***$P < 0.001$, **$P < 0.01$, *$P < 0.05$, and data are the mean ± SEM from at least three independent experiments.

Figure S11. VER155008 exhibits synergetic effect with BRAF inhibitor on inhibition of metastatic potential.

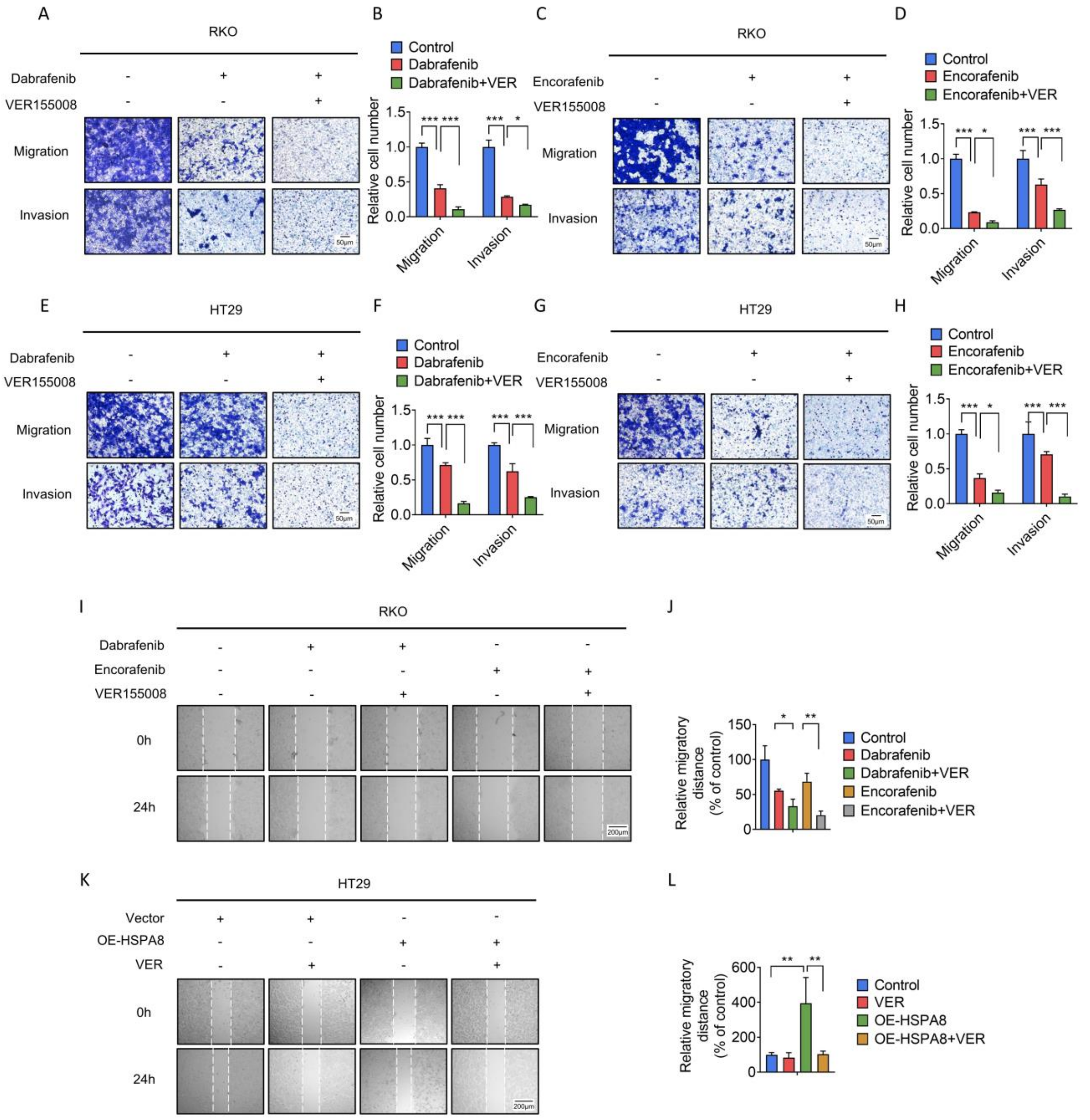

Figure S11. VER155008 exhibits synergetic effect with BRAF inhibitor on inhibition of metastatic potential. (A-H) Transwell assays of RKO and HT29 cells treated with Dabrafenib or Encorafenib, combined with or without VER155008. Scale bar: 50 μm. (I-L) Wound healing assay showing the migration of RKO and HT29 cells treated with Dabrafenib or Encorafenib, combined with or without VER155008. Scale bar: 200 μm. ***$P < 0.001$, **$P < 0.01$, *$P < 0.05$, and data are the mean ± SEM from at least three independent experiments.
